# Human pluripotent stem cell-derived ectomesenchymal stromal cells promote more robust functional recovery than umbilical cord-derived mesenchymal stromal cells after hypoxic-ischaemic brain damage

**DOI:** 10.7150/thno.57234

**Published:** 2022-01-01

**Authors:** Jiawei Huang, Kin Pong U, Fuyuan Yang, Zeyuan Ji, Jiacheng Lin, Zhihui Weng, Lai Ling Tsang, Tobias D Merson, Ye Chun Ruan, Chao Wan, Gang Li, Xiaohua Jiang

**Affiliations:** 1 School of Biomedical Sciences, Faculty of Medicine, The Chinese University of Hong Kong, Hong Kong SAR, PR China.; 2 Department of Orthopaedics and Traumatology, Faculty of Medicine, The Chinese University of Hong Kong, Hong Kong SAR, PR China.; 3 School of Biomedical Sciences Core Laboratory, Shenzhen Research Institute, The Chinese University of Hong Kong, Shenzhen, PR China.; 4 Sichuan University - The Chinese University of Hong Kong Joint Laboratory for Reproductive Medicine, West China Second University Hospital, Sichuan University, Chengdu 610041, Sichuan, China.; 5 Australian Regenerative Medicine Institute, Monash University, Clayton, VIC, Australia.; 6 Department of Biomedical Engineering, Faculty of Engineering, The Hong Kong Polytechnic University, Hong Kong, China.

**Keywords:** HIE, ectomesenchymal stromal cells, brain damage, regeneration, paracrine, ERK

## Abstract

**Aims:** Hypoxic-ischaemic encephalopathy (HIE) is one of the most serious complications in neonates and infants. Mesenchymal stromal cell (MSC)-based therapy is emerging as a promising treatment avenue for HIE. However, despite its enormous potential, the clinical application of MSCs is limited by cell heterogeneity, low isolation efficiency and unpredictable effectiveness. In this study, we examined the therapeutic effects and underlying mechanisms of human pluripotent stem cell-derived ectomesenchymal stromal cells (hPSC-EMSCs) in a rat model of HIE.

**Methods:** hPSC-EMSCs were induced from either human embryonic stem cells or induced pluripotent stem cells. Stem cells or the conditioned medium (CM) derived from stem cells were delivered intracranially or intranasally to neonatal rats with HIE. Human umbilical cord-derived MSCs (hUC-MSCs) were used as the therapeutic comparison control and phosphate-buffered saline (PBS) was used as a negative control. Lesion size, apoptosis, neurogenesis, astrogliosis and microgliosis were evaluated. The rotarod test and Morris water maze were used to determine brain functional recovery. The PC-12 cell line, rat primary cortical neurons and neural progenitor cells were used to evaluate neurite outgrowth and the neuroprotective and neurogenesis effects of hPSC-EMSCs/hUC-MSCs. RNA-seq and enzyme-linked immunosorbent assays were used to determine the secretory factors that were differentially expressed between hPSC-EMSCs and hUC-MSCs. The activation and suppression of extracellular signal-regulated kinase (ERK) and cAMP response element-binding protein (CREB) were characterised using western blotting and immunofluorescent staining.

**Results:** hPSC-EMSCs showed a higher neuroprotective potential than hUC-MSCs, as demonstrated by a more significant reduction in lesion size and apoptosis in the rat brain following hypoxia-ischaemia (HI). Compared with PBS treatment, hPSC-EMSCs promoted endogenous neurogenesis and alleviated astrogliosis and microgliosis. hPSC-EMSCs were more effective than hUC-MSCs. hPSC-EMSCs achieved a greater recovery of brain function than hUC-MSCs and PBS in rats with HIE. CM derived from hPSC-EMSCs had neuroprotective and neurorestorative effects *in vitro* through anti-apoptotic and neurite outgrowth- and neurogenesis-promoting effects. Direct comparisons between hPSC-EMSCs and hUC-MSCs revealed the significant enrichment of a group of secretory factors in hPSC-EMSCs, including nerve growth factor (NGF), platelet-derived growth factor-AA and transforming growth factor-β_2_, which are involved in neurogenesis, synaptic transmission and neurotransmitter transport, respectively. Mechanistically, the CM derived from hPSC-EMSCs was found to potentiate NGF-induced neurite outgrowth and the neuronal differentiation of NPCs via the ERK/CREB pathway. Suppression of ERK or CREB abolished CM-potentiated neuritogenesis and neuronal differentiation. Finally, intranasal delivery of the CM derived from hPSC-EMSCs significantly reduced brain lesion size, promoted endogenous neurogenesis, mitigated inflammatory responses and improved functional recovery in rats with HIE.

**Conclusion:** hPSC-EMSCs promote functional recovery after HI through multifaceted neuromodulatory activities via paracrine/trophic mechanisms. We propose the use of hPSC-EMSCs for the treatment of HIE, as they offer an excellent unlimited cellular source of MSCs.

## Introduction

Hypoxic-ischaemic encephalopathy (HIE) is one of the most serious complications in neonates and infants, with an incidence of 3 per 1,000 live births in developed countries. Although the exact cause of HIE has not been identified, conditions such as the mismanagement of high-risk pregnancies, uterine rupture and delayed emergency C-sections are known to contribute to the development of HIE 1. Notably, the brains of neonates and infants are particularly vulnerable to hypoxia-ischaemia (HI) challenge because of their high energy demands. When the levels of oxygen and glucose delivered to the brain can no longer meet its metabolic demands, a sequence of biochemical events is triggered that leads to global brain injury 2. Lifelong consequences, such as cerebral palsy (CP), epilepsy, vision or hearing loss and cognitive impairments, can be devastating to the patients. Current therapeutic interventions for neonates and infants with HIE are limited and predominantly focus on the prevention of apoptosis and necrosis during the early phases of brain injury 3. Indeed, therapeutic hypothermia is the only recognised effective treatment for term infants with HIE of low severity 4. Hypothermia, however, has not been demonstrated to improve outcomes related to severe HIE. However, emerging evidence shows that, besides prevention and stabilisation, repair and regeneration may be critical to achieve the long-term goal of improving functional recovery of the affected brain after HI. Unfortunately, hypothermia can neither replace lost neurons, nor promote regeneration, which are critical for brain repair. Therefore, alternative treatment strategies, such as those involving stem cells, are needed to treat severe HIE.

Over the past decade, stem cell therapy has emerged as a novel approach to target the multiple pathological processes involved in HIE. Compared with other sources of stem cells, mesenchymal stromal cells (MSCs) exhibit many favourable characteristics, including accessibility, lack of ethical issues and immunological compatibility 5. Indeed, a variety of exogenous MSCs have been observed to protect against global and local neuro-inflammatory cascades triggered by HI, activate anti-apoptotic mechanisms, differentiate into neurons to replace damaged tissues and generate the trophic milieu necessary for endogenous repair 6. More importantly, several recent clinical studies have demonstrated that MSC transplantation at either the primary or later injury stages can accelerate functional recovery, reduce disability and improve the quality of life of patients with CP 7-9. Although the cellular and molecular mechanisms underlying the beneficial effects of MSCs are largely unknown, it has become evident that the reparative activities of MSCs are mainly associated with their paracrine effects 10. Interestingly, MSCs secrete a plethora of growth factors and cytokines known to influence neuronal survival, differentiation, neurite outgrowth and immunomodulation 11, 12. Moreover, the direct transplantation of secretory factors derived from MSCs activates genes that are involved in neurogenesis, axonal sprouting, angiogenesis and immunomodulation in the host animals 13-15. Cumulatively, these studies strongly suggest that MSCs secrete bioactive factors that promote endogenous repair and regenerative processes in the ischaemic brain.

Despite their attractive therapeutic potential, the clinical application of MSCs has been limited by several scientific hurdles, including the heterogeneity of the cells, low isolation efficiency and the loss of stem cell function after cultivation. In addition, MSCs isolated from adult tissues have a limited life span *in vitro* due to replicative senescence 16, 17. Higher-passage MSCs are also more predisposed to trigger innate immune attacks upon transplantation in humans 18. On the other hand, previous studies have indicated that different sources of MSCs are likely to have distinct secretion profiles 19, 20. For instance, exposure of primary cultures of hippocampal neurons to the conditioned medium (CM) of adipose tissue-derived MSCs and human umbilical cord perivascular stromal cells results in different effects on cell proliferation and metabolic activity 21. MSCs derived from Wharton's jelly secrete more factors related to angiogenesis and neurogenesis than those secreted by BM-MSCs 22. Given such differences in the secretory profiles of MSCs obtained from different tissue sources, the ideal source of MSCs will differ for specific applications and must be determined accordingly.

Ectomesenchymal stromal cells (EMSCs), which are derived from the cranial neural crest (NC) generated at the junction between the anterior neuroectoderm and the surface ectoderm 23, are a unique type of MSC with both neural and mesenchymal traits. Recent studies have shown that EMSCs are very effective at treating neurological diseases, such as ischaemic stroke, probably due to their ectodermal origin and neural propensity 24, 25. Nevertheless, despite their attractive therapeutic potential, the use of EMSCs in tissue regeneration has been limited by their scarcity and heterogeneity in adult tissues, including dental pulp and olfactory mucosa. PSC-derived MSCs are able to be expanded extensively and have enhanced therapeutic potential compared with MSCs from other sources 26-28, due to their 'rejuvenated' phenotypes. Thus, infinitely expandable PSCs may provide an unlimited source of EMSCs, thereby improving production efficiency and reproducibility and eliminating the need for potentially risky and invasive procedures. In this regard, PSC-EMSCs may be promising donor cells for the treatment of HIE. In this study, for the first time, we investigated the therapeutic effects and underlying mechanisms of hPSC-EMSCs in a rat model of HIE.

## Methods

### Cell Culture

#### Differentiation of hEMSCs from hPSCs

HESCs (H7 and H9; WiCell Research Institute, Madison, WI, USA) and iPSCs, derived from bone marrow CD34^+^ cells (American Type Culture Collection [ATCC], ACS1031) of an Asian female (46, XX, 27 years old), were cultured as previously described 29. Briefly, cells were maintained in Geltrex-coated culture plates and StemFlex medium (Gibco, Life Technologies, Carlsbad, CA, USA). The medium was changed every day and the cells were routinely passaged at a 1:6 (vol/vol) ratio on Geltrex-coated plates every 3-4 days after treatment with Accutase (Gibco).

HESCs or iPSCs were first induced to differentiate into human NC progenitor cells (hNCPCs). Briefly, 90% confluent hESCs/iPSCs were dissociated into single cells by treating them with Accutase at 37 *°*C for 5 min. Single cells were seeded on Geltrex-coated plates in StemFlex medium at a density of 9.2 × 10^4^ cells per cm^2^. The next day, the medium was changed to NC medium 29 to initiate NC differentiation. After 16 days of differentiation in NC medium, the NC identity of the differentiating cells was confirmed using flow cytometry (p75^+^ and HNK1^+^). To differentiate hNCPCs into EMSCs, 90% confluent hNCPCs were dissociated into single cells by treating them with Accutase at 37 *°*C for 1-3 min. The single cells were then seeded in NC medium on Geltrex-coated plates at a density of 6.5 × 10^4^ cells per cm^2^. After 24 h, the medium was replaced with MSC medium (α-minimal essential medium, 10% foetal bovine serum [FBS], 100 U/mL penicillin-streptomycin). After 15 days of differentiation, the hPSC-EMSCs were analysed for surface markers using flow cytometry (CD73^+^, CD44^+^, CD105^+^ and CD90^+^). hPSC-EMSCs at passages 3-10 were used in this study. The identity of the hNCPCs was confirmed by their multi-lineage differentiation. To induce myofibroblast differentiation, Dulbecco's modified Eagle's medium (DMEM) with 10% FBS was added and the medium was changed every other day. The cells were stained for smooth muscle actin on day 30. To induce peripheral neuronal differentiation, the hNCPC medium was changed to DMEM/F12 supplemented with 1× N2 supplement, heregulin (20 ng/mL), fibroblast growth factor 2 (10 ng/mL) and cyclic adenosine monophosphate (cAMP, 5 µM). The cells were fixed and stained with peripherin on day 15. To induce osteogenic differentiation, hPSC-EMSCs were seeded onto 6-well plates at a density of 4 × 10^5^ cells per well and 5 nM of dexamethasone (Sigma-Aldrich, St Louis, MO, USA; D4902), 50 μg/mL of ascorbic acid 2-phosphate (Sigma-Aldrich, A8960) and 10 mM of glycerol 2-phosphate (Sigma-Aldrich, G9891) were added to the culture medium. The cells were fixed on day 16 and stained with Alizarin Red.

#### hUC-MSCs

The use of human umbilical cords for MSC isolation was approved by the Joint Chinese University of Hong Kong-North Territories East Cluster Clinical Research Ethics Committee (ethical approval code: CRE-2011.383 and CRE-2015.018), as described in our previous study 30. The cell culture reagents were purchased from Gibco. Cells were cultured at 37 *°*C with 5% CO_2_ in MSC medium. After reaching 90% confluence, hUC-MSCs were passaged using 0.05% trypsin and re-plated at a density of 10,000 cells/cm^2^ in T175 or T75 flasks. Three hUC-MSC lines (hUC009, hUC011 and hUC013) at passage 3-10 were used in this study.

#### Flow cytometry

MSCs were characterised using the Human MSC Analysis Kit (BD Biosciences, San Jose, CA, USA) in accordance with the manufacturer's instructions. Briefly, cells were detached and washed in staining buffer and centrifuged at 1,000 × *g* for 3 min. The cell pellets were re-suspended in the appropriate primary antibody (anti-CD105, anti-CD90, anti-CD73 or anti-CD44) solution at a dilution of 1:20 in staining buffer and incubated for 30 min on ice. The cells were then washed twice, re-suspended in staining buffer and analysed using a BD LSR Fortessa Cell Analyzer (BD Biosciences) and FlowJo v10.2 software.

#### Preparation of CM

The CM of hPSC-EMSCs/hUC-MSCs was collected as previously described, with minor modifications 31. MSCs were seeded in 75 cm^2^ flasks and incubated in complete culture medium. When the cells reached 80-90% confluency, they were rinsed three times with PBS and the medium was replaced with 20 mL of serum-free medium. After 48 h, the serum-free culture medium was transferred to a centrifuge tube and centrifuged at 2,000 × *g* for 10 min to remove cell debris. For *in vivo* studies, 30 mL of CM was concentrated and dialysed with double-distilled water at 4 °C to remove ions and molecules below 1.0 kDa, and then freeze-dried. Protein concentration was determined using a Pierce™ BCA Protein Assay Kit (Thermo Fisher Scientific, Waltham, MA, USA), with results obtained by measuring the absorbance at 562 nm.

#### PC-12 cells

PC-12 cells were obtained from the ATCC. The cells were cultured in RPMI 1640 medium supplemented with 10% horse serum, 5% FBS and 100 U/mL penicillin-streptomycin at 37 *°*C in a water-saturated atmosphere with 5% CO_2_. To induce differentiation, PC-12 cells were incubated with 50 ng/mL of nerve growth factor (β-NGF, Gibco) and 1% FBS for 4 days.

#### Isolation of rat NPCs or primary neurons

Rat (r)NPCs were isolated in accordance with a previously established protocol 32. In brief, postnatal day 1 (PN1) Sprague-Dawley (SD) rat hippocampi were dissected and isolated in ice-cold DMEM. The hippocampi were then digested and dissociated in Accutase for 10 min at 37 *°*C with trituration for 10 s every 2 min. Tissue fragments and cells were centrifuged at 1,200 rpm for 5 min and the resulting supernatant was removed. The cells were re-suspended in NPC medium (DMEM/F12 supplemented with 2 mM of L-glutamine, 2% B-27, 5 µg/mL of heparin sulphate, 20 ng/ mL of basic fibroblast growth factor [bFGF], 20 ng/mL of epidermal growth factor and 100 U/mL of penicillin-streptomycin), seeded on Geltrex-coated vessels and cultured for 5 days. Attached cells were detached after 5 days using Accutase, seeded on bovine gelatin-coated vessels and cultured for 5-7 days in NPC medium. The attached cells were removed and floating neurospheres collected and dissociated using Accutase and trituration. The cells were centrifuged at 1,200 rpm for 5 min, re-suspended in NPC medium and seeded on Geltrex-coated vessels.

Primary neurons were isolated from the cortices of PN1 SD rats, as described previously 33. Briefly, the cortices were dissected, mechanically minced and dissociated by treatment with 0.25% trypsin at 37 *°*C for 20 min. After centrifugation at 1,000 rpm for 5 min, single cell suspensions were prepared in neurobasal medium (Gibco) with 10% FBS and 100 U/mL of penicillin-streptomycin and incubated at 37 *°*C in a humidified atmosphere of 5% CO_2_ for 24 h. After attachment, primary neurons were cultured in neurobasal medium with 2% B-27 (Gibco) and 2 mM L-glutamine (Gibco) for 4 days before conducting further experiments.

#### Neuronal differentiation of rNPCs

rNPCs were seeded onto Geltrex-coated chamber slides at a density of 2,000 cells per chamber and grown to 60% confluency in rNPC medium. To differentiate the rNPCs into neurons, the rNPC medium was replaced with neuron-priming medium (DMEM/F12 supplemented with 2 mM of L-glutamine, 2% B-27, 5 µg/mL of heparin sulphate, 20 ng/mL of bFGF and 100 U/mL of penicillin-streptomycin) for 48 h. The medium was then replaced with neuronal differentiation medium (DMEM/F12 supplemented with 2 mM of L-glutamine, 2% B-27, 20 ng/mL bFGF, 5 µg/mL of heparin sulphate, 20 ng/mL of brain-derived neurotrophic factor and 100 of U/mL penicillin-streptomycin). To determine the effects of CM on rNPC neuronal differentiation, DMEM/F12 basal medium was replaced with hPSC-EMSC-derived CM in neuron-priming and neuronal differentiation media and supplemented with the same growth factors.

### *In vitro* oxygen-glucose deprivation and reperfusion model

To establish an oxygen-glucose deprivation and reperfusion (OGD/R)-induced cell injury model, PC-12 cells or primary neurons were transferred to serum- and glucose-free medium and incubated under 2% O_2_, 93% N_2_ and 5% CO_2_ for 4 h in a Galaxy incubator (Eppendorf, Hamburg, Germany). After OGD, the cells were transferred to a medium with glucose and returned to normoxic conditions maintained at 37 *°*C and 5% CO_2_ for 24 h for re-oxygenation. The required CM was added 16 h before OGD and maintained until the end of the re-oxygenation.

### MTS cell viability assay

PC-12 cells were seeded onto 96-well plates at a density of 30,000 cells per well and treated with OGD/R. After 4 h of OGD and 24 h of re-oxygenation, the cell viability was determined using the Cell Titer96^®^ Aqueous cell proliferation assay (Promega, Madison, WI, USA). The absorbance was measured at 490 nm using a microplate reader.

### Terminal transferase-mediated dUTP nick-end labelling assay

To measure apoptosis, terminal transferase-mediated dUTP nick-end labelling (TUNEL) staining was performed on PC-12 cells at 24 h post-OGD/R, in accordance with the manufacturer's instructions (Roche, Basel, Switzerland). Cell nuclei were stained with 4',6-diamidino-2-phenylindole (DAPI). Apoptosis was quantified as the percentage of TUNEL/DAPI-positive cells, which were counted using ImageJ software (National Institutes of Health, Bethesda, MD, USA).

### Neurite outgrowth assessment

To measure neurite outgrowth, cells were fixed, processed and imaged using an IX83-ZDC automated inverted microscope (Olympus, Tokyo, Japan) under the 40× objective. The coverslips were scanned from left to right, and 8-10 fields were randomly selected. For each field, neurites were traced and measured using ImageJ software, and at least 30 cells from three independent experiments were scored for each condition. A cell was considered to be neurite-bearing if it contained at least one neuronal process that was longer than the cell body. For inhibitor or activator studies, KG501 (1 μM), U0126 (10 μM) or forskolin (10 μM) was added to the PC-12 cell culture medium concurrently with NGF (50 ng/mL) and the cells were incubated for the indicated times.

### RNA sequencing

RNA-seq library preparation and sequencing were performed by the Beijing Genomics Institute (Shenzhen, China). Briefly, total RNA from treated samples was extracted using TRIzol reagent (Invitrogen, Carlsbad, CA, USA). mRNA samples were prepared for the RNA-seq analysis using the Illumina TruSeq RNA Sample Prep Kit V2 (Illumina, San Diego, CA, USA) in accordance with the manufacturer's instructions. To construct the final cDNA libraries, universal adapters were ligated to the cDNA fragments, followed by PCR amplification. The quality of the sequencing library was tested using an Agilent 2100 bioanalyzer (Agilent, Santa Clara, CA, USA). To enrich cDNA in the library, PCR was performed to selectively amplify fragments with adapter molecules on both ends. The established cDNA libraries were then sequenced on the HiSeq2000 platform (TruSeq SBS KIT-HS V3, Illumina) with a paired-end sequencing length of 90 bp. The levels of gene expression were determined and differentially expressed genes (DEGs) were identified using the method described by Audic and Claverie 34. Gene expression levels were calculated using the reads per kilobase of transcript, per million mapped reads method. In cases where more than one transcript was found for a gene, the longest read was used to calculate its expression level and coverage. Significantly DEGs were determined at a false discovery rate threshold ≤ 0.001 and an absolute log2 ratio value of ≥ 1.0. A gene heatmap and correlation plot were generated via an online platform (http://www.bioinformatics.com.cn/). Gene set enrichment analysis was performed via the Metascape online analysis tool (http://metascape.org/) 35.

### Enzyme-linked immunosorbent assays

Enzyme-linked immunosorbent assays (ELISAs) were performed in accordance with the protocols outlined in the Human β Nerve Growth Factor PicoKine™ ELISA Kit, Human TGF-β2 PicoKine™ ELISA Kit, Human PDGF-AA PicoKine™ ELISA Kit and Human Rantes (CCL5) PicoKine^TM^ ELISA Kit (Boster Bio, Pleasanton, CA, USA).

### Reverse transcription quantitative PCR

Total RNA was isolated using TRIzol reagent and reverse transcribed to cDNA. Triplicate samples containing the appropriate primers were analysed by reverse transcription quantitative PCR (RT-qPCR) using SYBR Green PCR Master Mix (Promega) in accordance with the manufacturer's instructions. The primers used for specific genes are presented in Table S1. Target gene expression levels were normalised to *GAPDH* expression levels. Relative gene expression was calculated using the 2^-ΔΔCt^ formula.

### Western blotting

After exposure to different treatments, cells were lysed and protein was extracted using radioimmunoprecipitation assay buffer (Pierce, Rockford, IL, USA) containing a protease inhibitor cocktail (Thermo Fisher Scientific). Protein concentrations were determined using the bicinchoninic acid assay (Bio-Rad, Richmond, CA, USA). Equal amounts of protein were separated using 10% sodium dodecyl sulphate-polyacrylamide gel electrophoresis and transferred to 0.2 μm polyvinylidene fluoride membranes (Millipore, Burlington, MA, USA), which were blocked in 5% non-fat milk for 1 h. The membranes were then incubated overnight at 4 *°*C with rabbit polyclonal anti-p-ERK (1∶1,000; Cell Signaling Technology, Danvers, MA, USA), rabbit monoclonal anti-p-CREB (1∶1,000, Cell Signaling Technology), rabbit monoclonal anti-CREB (1:1,000, Cell Signaling Technology), rabbit monoclonal anti-ERK (1∶1,000, Cell Signaling Technology) or mouse monoclonal anti-β-actin antibodies (1∶1,000; Santa Cruz Biotechnology, Dallas, TX, USA). After washing with TBS/T (TBS with 0.05% Tween 20), the membranes were incubated with the appropriate horseradish peroxidase-conjugated secondary antibodies (1∶5,000, Invitrogen) at room temperature for 1 h. The antigen-antibody complexes were then detected using an ECL reagent kit (GE Healthcare), visualised using an X-ray film processor and quantified using ImageJ software. The antibodies used for the western blotting are listed in Table S2.

### Animal ethics, selection and welfare

*In vivo* studies were performed with the approval of the Animal Experimentation Ethics Committee of the Chinese University of Hong Kong (ethics number: 19-148-ITF). Pregnant SD rats were individually housed in cages and fed standard laboratory chow *ad libitum*. The health status of the rats was monitored daily, and no obvious behavioural phenotypes were observed. Rat pups were randomly assigned to experimental groups after HI insult. The sample size for all of the animal experiments was determined on the basis of previous experience, pilot experiments and power calculations made based on the following criteria: a power of 80% and a significance level < 0.05. The sample sizes for each experiment are included in the figure legends. The researchers involved in the biochemical and behavioural analyses were blinded to the group allocation for the duration of the study.

### Animal model of HIE

HI was induced as previously described 36. Briefly, PN7 SD rat pups (14 to 16 g) were anaesthetised and exposed to surgical ischaemia on a heating pad set to 37 *°*C. The right common carotid artery was exposed, isolated from the nerve and vein and permanently doubly ligated with 6-0 surgical silk. After 1 h of recovery, the pups were transferred to a hypoxic chamber on a heating pad and subjected to a humidified mixture of 8% O_2_ and 92% N_2_ for another 2 h. The surface and core body temperatures of the animals were monitored during hypoxia, and were maintained at 32-33 °C and 36.5-37 °C, respectively, using a homeothermic monitoring system (Harvard Apparatus, Holliston, MA, USA). Using these experimental conditions, the brain damage pattern obtained was consistent with that reported in other studies that used the Rice-Vannucci HI model, showing moderate to severe brain damage in the cerebral cortex, hippocampus and striatum in the majority of the animals. Sham animals were subjected to the same experimental procedure, but without HI insult. Animals were excluded only if mortality occurred during surgery or before the treatment was administered.

### Intracranial delivery of hPSC-EMSCs or hUC-MSCs

On the third day after HI insult (PN10), 2 × 10^5^ hUC-MSCs or hPSC-EMSCs in 5 μL of PBS or vehicle were infused into the ipsilateral hemisphere at 2 mm caudal to the bregma, 1.5 mm right from the midline and 3 mm below the dural surface, while the pups were under isoflurane. For cell tracing experiments, hPSC-EMSCs were labelled with the red fluorescent dye, PKH26 (Sigma-Aldrich, MINI126), or transduced with plenti-GFP as previously described 30, and rat bone marrow MSCs were isolated from GFP-rats 37.

### Intranasal delivery of CM derived from hPSC-EMSCs

For intranasal delivery, one set of animals was randomly designated as the control group and received PBS. The other set of animals was administered CM derived from hPSC-EMSCs. The CM was re-suspended in PBS and administered using a pipette to alternating nares every 5 min in 1 μL (3 μg/μL) drops from PN9. Over 60 min, a total volume of ~12 μL of PBS or CM was administered each day (36 μg/day). The animals were treated for 7 consecutive days, after which one set of animals was sacrificed on PN20 for histological studies and another set of animals was used for behavioural tests from PN30 to PN35.

### Animal grouping

Two hundred and five rat pups were evaluated in this study. In general, mortality (10-15%) occurred after the HIE procedure between PN7 and 10. The severity of brain damage in this model was determined by several key factors including the duration of surgery, the amount of rest time before hypoxia, the hypoxia period and the degree of temperature control. The relatively higher mortality rate we observed in this study was probably due to the shorter rest time (1 h) between surgery and hypoxic challenge compared to other studies (2-8 h) 38-40. Fifteen pups were used for the cell tracing experiments, and 88 pups were used for stem cell treatment and the mechanistic study. Animals were grouped into sham (n = 12), PBS (n = 24), hUC-MSC (n = 26) and hPSC-EMSC (n = 26) groups and were sacrificed on PN16 and PN20 for histological and molecular studies, respectively. For the stem cell treatment and behavioural analyses, 62 pups were divided into sham (n = 16), PBS (n = 15), hUC-MSC (n = 15) and hPSC-EMSC (n = 16) groups. For the CM treatment and mechanistic study, 18 pups were included in the experiments. Animals were grouped into sham (n = 6), PBS (n = 6) and hPSC-EMSC (n = 6) groups and were sacrificed on PN20 for histological studies. For the CM treatment and Morris water maze (MWM) tests, 22 pups were divided into sham (n = 8), PBS (n = 6) and EMSC-CM (n = 8) groups.

### Haematoxylin and eosin staining and lesion analysis

Brains were collected at PN16 and PN20 and fixed in optimum cutting temperature compound (Tissue-tek, Sakura Finetek, Tokyo, Japan) solution. Coronal sections (10 μm) were cut every 400 μm. Three cryosections from each brain tissue sample were washed with PBS for 5 min and then dipped in water for 2 min. After rehydration, the sections were stained with haematoxylin for 5 min and differentiated in acid ethanol for 3 min. The sections were then washed in running tap water for 2 min, blued in tap water for 10 min and stained with eosin Y solution for 1-2 min. The sections were dehydrated and cleared with xylene twice and finally, mounted using histological mounting medium. The lesions were imaged using an Olympus stereo microscope (SZX16) and quantified based on the ratio of the ipsilateral (injured) side to the contralateral side of the brain using ImageJ software. Three cryosections from each brain tissue sample were analysed by two independent researchers (Huang J and U KP).

### Immunofluorescence staining

Cells and frozen brain sections were fixed with 4% paraformaldehyde (Sigma-Aldrich) and permeabilised with 0.1% Triton X-100 for 10 min. After blocking with 3% bovine serum albumin, the fixed cells or tissues were incubated with appropriate primary antibodies at 4 *°*C overnight. The slides were then rinsed and incubated with fluorescein isothiocyanate/tetramethylrhodamine-conjugated secondary antibodies (Invitrogen) at room temperature for 1 h in the dark. The cells or tissues were visualised using an Olympus IX83 inverted microscope or a Leica TCS SP8 inverted confocal microscope (Leica, Wetzlar, Germany) and analysed using Image J software. For all of the quantitative analyses, images were collected using the same acquisition parameters to facilitate fluorescence intensity comparisons between groups. For brain tissue staining, 3-4 sections were assessed per antibody in each rat. In all of the studies, a region of interest (ROI) was chosen based on the anatomical structures of the cortex and/or subventricular zone (SVZ) and the hippocampus based on DAPI staining. The number of cells was normalised to the surface area of each ROI. The mean fluorescence intensity of positive signals per cell was calculated and presented as arbitrary units. Spots were designated as positive cells when their signals were above a set threshold signal intensity, and this threshold was applied across all of the analyses. Specifically, analyses of cleaved caspase-3, GFAP, Iba-1 and vimentin were performed in three sections in the cortex adjacent to the lesion (6 days post-injection [dpi], bregma -1.7 mm~-2.1 mm; 10 dpi and PN20, bregma -2.0 mm~-2.4 mm). The intensity of nestin and Sox2 staining was evaluated in 3-4 consecutive sections and within 450 μm from the SVZ to include predominant newborn cells that had migrated from the SVZ (6 dpi, bregma -1.7 mm~-2.1 mm; 10 dpi and PN20, bregma -2.0 mm~-2.4 mm). Analyses of DCX, p-ERK and p-CREB were performed in the hippocampi (bregma -2.0 mm~-2.4 mm) in three consecutive sections. For the cell line studies, the percentage of positive cells, defined as the number of positive cells/DAPI-positive cells, was calculated. Five fields were analysed per slide under a microscope and no less than 200 cells were analysed for quantification. The experiments were performed three times and each experiment was set up with triplicate samples. Immunofluorescence staining data were analysed by two independent researchers (Huang J and U KP). The antibodies used are listed in Table S2.

### Functional outcomes

#### Rotarod test

Rats were subjected to a rotarod test (Ruanlong, BW-ZH300, China) on the 11^th^ through to the 13^th^ day after stem cell transplantation to evaluate their motor and coordination performance. The animals were placed on a rotating cylinder for 60 s, the speed of which increased from 5 rpm to 32 rpm. During that time, each rat that was able to stay on the cylinder was recorded. Three interval trials were performed on each day. The mean time taken for the rats to fall off the cylinder, the speed of the rotating rod when the rats fell and the number of rats that fell off were recorded for each group.

#### Morris water maze test

Eleven days after stem cell transplantation (PN21), the rats were subjected to the MWM task to evaluate their spatial learning and memory functions, as previously described 41. For the CM treatment, the rats were subjected to MWM on PN30-35. Briefly, on the first day of training, the rats were exposed to a visible platform to assess their visual capability. On the 2^nd^ through to the 5^th^ day, the rats were continuously trained using an invisible platform to enhance their learning and memory functions. The average time taken from escape to locating the platform was recorded from the 1^st^ through to the 5^th^ day. On the 6^th^ day, a probe trial was performed without an escape platform, and the time that the rats spent in the target quadrant during the 60 s test period was recorded.

#### Statistical analysis

Prism v6.01 (GraphPad Software, San Diego, CA, USA) was used to perform the statistical analyses. A two-tailed Student's t-test was used for comparisons of two groups of samples with normal distributions. A one-way analysis of variance (ANOVA) with Tukey's multiple comparison *post hoc* test was used for comparisons of more than two groups, whereas a two-way ANOVA with Tukey's *post hoc* test was used when two independent variables were assessed. The presented *p* value indicates the *post hoc* test value only if an overall statistically significant difference was seen after performing an ANOVA. Before performing an ANOVA or Student's t-test, normality was determined using a D'Agostino-Pearson normality test (omnibus K^2^). In each group, data were analysed in triplicate, unless otherwise indicated, and are presented as the mean ± standard error of the mean.

## Results

### hPSC-EMSCs exhibit enhanced neuroprotective effects compared with hUC-MSCs in rats with HIE

Either hESCs (H7 and H9) or human iPSCs (Figure S1A) were differentiated into hNCPCs, as described by Menendez et al. 2013 29 (Figure S1B). After 16 days of differentiation, more than 90% of the cells were found to be HNK1^+^/p75^+^ (Figure S1C, S2A) and able to differentiate into peripheral neurons, myofibroblasts and MSCs, thus confirming their identity as hNCPCs (Figure S1D). The hNCPCs were further differentiated into hPSC-EMSCs, resulting in changes in their cellular morphology from cuboidal, epithelial-like cells to fibroblastic cells (Figure S1B). More than 95% of the hPSC-EMSCs expressed CD105, CD44 and CD73 and were negative for the hematopoietic markers CD34, CD11, CD19, CD45 and HLA-DR. The only difference observed was in CD90 expression. Approximately 98% of the hESC-EMSCs expressed CD90, whereas approximately 85% of the hiPSC-EMSCs expressed CD90 (Figure S1E, S2B).

**Figure 1:**
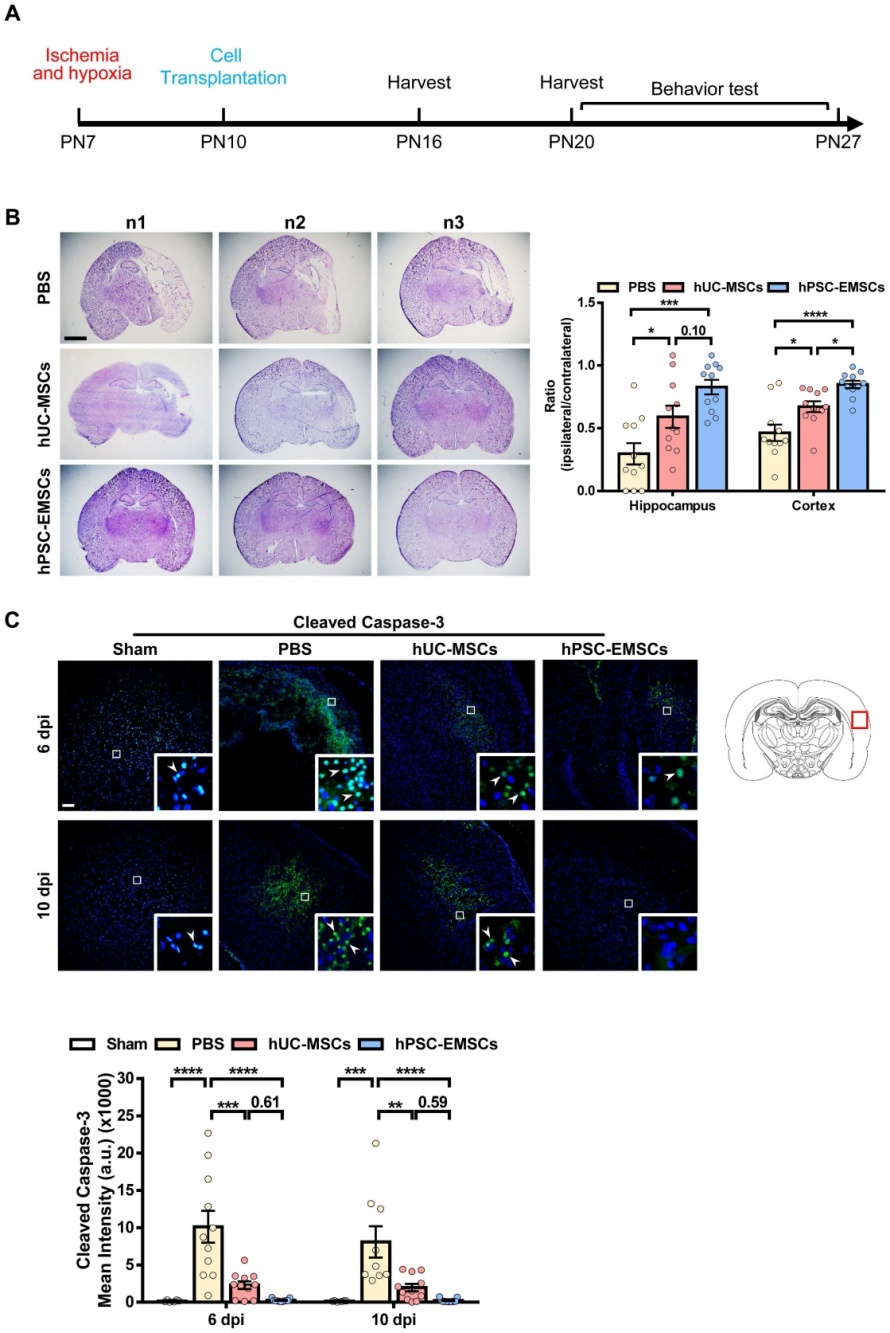
** hPSC-EMSCs reduce brain tissue damage more profoundly than hUC-MSCs in the rats with HIE.** The third day after HI (PN10), 2 × 10^5^ hUC-MSCs or hPSC-EMSCs (derived from H7 or iPSCs) in 5 µL of PBS or vehicle were infused into the ipsilateral hemisphere. **A)** The time line of HIE model construction, MSC transplantation, tissue harvest and behavior tests; **B)** Bright field images and quantification of H&E-stained ipsilateral/ contralateral brain cryosections collected from PBS-, hUC-MSC- and hPSC-EMSC-transplanted rats, scale bar: 2.5 mm. Quantification data represent mean ± SEM (6 dpi, PBS: n = 11, hUC-MSCs: n = 11, hPSC-EMSCs: n = 11; 10 dpi, PBS: n = 9, hUC-MSCs: n = 11, hPSC-EMSCs: n = 11); **C)** Representative immunofluorescent staining images of cleaved caspase-3 in the cortex. Focused area in the brain cortex is shown with a magnified view (inset) illustrating cleaved caspase-3 positive cells (green, arrowhead). The average intensity of cleaved caspase-3 was measured in three coronal sections (6 dpi, bregma -1.7, -1.8 and -1.9 mm; 10 dpi, bregma -2.0, -2.1 and -2.2 mm), scale bar: 100 µm. Quantification data represent mean ± SEM (6 dpi, sham: n = 6, PBS: n = 11, hUC-MSCs: n = 11, hPSC-EMSCs: n = 11; 10 dpi, sham: n = 6, PBS: n = 9, hUC-MSCs: n = 11, hPSC-EMSCs: n = 11). *, **, ***, **** represent p < 0.05, 0.01, 0.001 and 0.0001 respectively by Tukey's *post-hoc* test when statistical significance by One-way ANOVA (*p* < 0.05) was obtained.

To determine whether hPSC-EMSCs have a more significant therapeutic effect on HIE, we established a rat neonatal HIE model and compared the therapeutic potential of hPSC-EMSCs to that of PBS and hUC-MSCs. Either hUC-MSCs or hPSC-EMSCs were transplanted into the ipsilateral side of rat brains at PN10 (3 days after HI insult, Figure 1A). To assess the presence of human cells in the rats after intracranial administration, we labelled the hPSC-EMSCs with PKH26. PKH26-positive cells were found at the injured cerebral cortex, hippocampus and lateral ventricle in the HIE group at 3 dpi. However, at 10 dpi, hPSC-EMSCs were barely detectable in any region of the brains of rats with HIE (Figure S3A). Consistently, a limited number of GFP-labelled hPSC-EMSCs were found in the rat brains at 10 dpi (Figure S3B). The low retention rate of the stem cells may be due to immune rejection, as a large number of rat MSCs still survived at 10 dpi (Figure S3C). While both hPSC-EMSC and hUC-MSC transplantation reduced lesion size in the cortex and hippocampus at 6 dpi, the hPSC-EMSCs exhibited a stronger effect than the hUC-MSCs in alleviating tissue loss. The ipsilateral/contralateral ratio of the cortex in the hPSC-EMSC-treated rats recovered to 0.849 ± 0.03 compared to 0.671 ± 0.044 in the hUC-MSC-treated rats and 0.463 ± 0.066 in the PBS-treated rats (Figure 1B). In the hippocampus, the ipsilateral/contralateral ratio in the hPSC-EMSC-treated rats recovered to 0.827 ± 0.058 compared to 0.591 ± 0.088 in the hUC-MSC-treated rats and 0.296 ± 0.085 in the PBS-treated rats. At 10 dpi, no differences were detected between the hUC-MSC- and PBS-treated groups, but hPSC-EMSCs significantly promoted repair in both the cortex and hippocampus (Figure S4B). More severe damage was also observed in the male rats, and hUC-MSCs/hPSC-EMSCs more effectively promoted repair in the male rats than in the female rats (Figure S4A, B). To further examine whether MSC transplantation prevents neuronal damage by suppressing apoptosis, the number of cleaved caspase-3-positive cells was determined in the perilesional area of the cortex. In the PBS-treated HIE brains, cleaved caspase-3-positive cells were present near the lesions. In addition to a reduction in lesion size, both sets of MSC-transplanted HIE brains exhibited a significant downregulation in the levels of cleaved caspase-3, although hPSC-EMSC transplantation mitigated the apoptotic response in a more pronounced manner (Figure 1C). At 10 dpi, almost no apoptotic cells were detected in the hPSC-EMSC-treated brains. Of note, while hUC-MSCs significantly alleviated the apoptotic response only in the female rats, hPSC-EMSCs showed markedly reduced apoptosis in both the male and female rats (Figure S4C). These results indicate that hPSC-EMSCs have a greater neuroprotective effect than hUC-MSCs after HIE.

### hPSC-EMSCs promote endogenous neurogenesis and alleviate inflammatory responses more effectively than hUC-MSCs in rats with HIE

**Figure 2:**
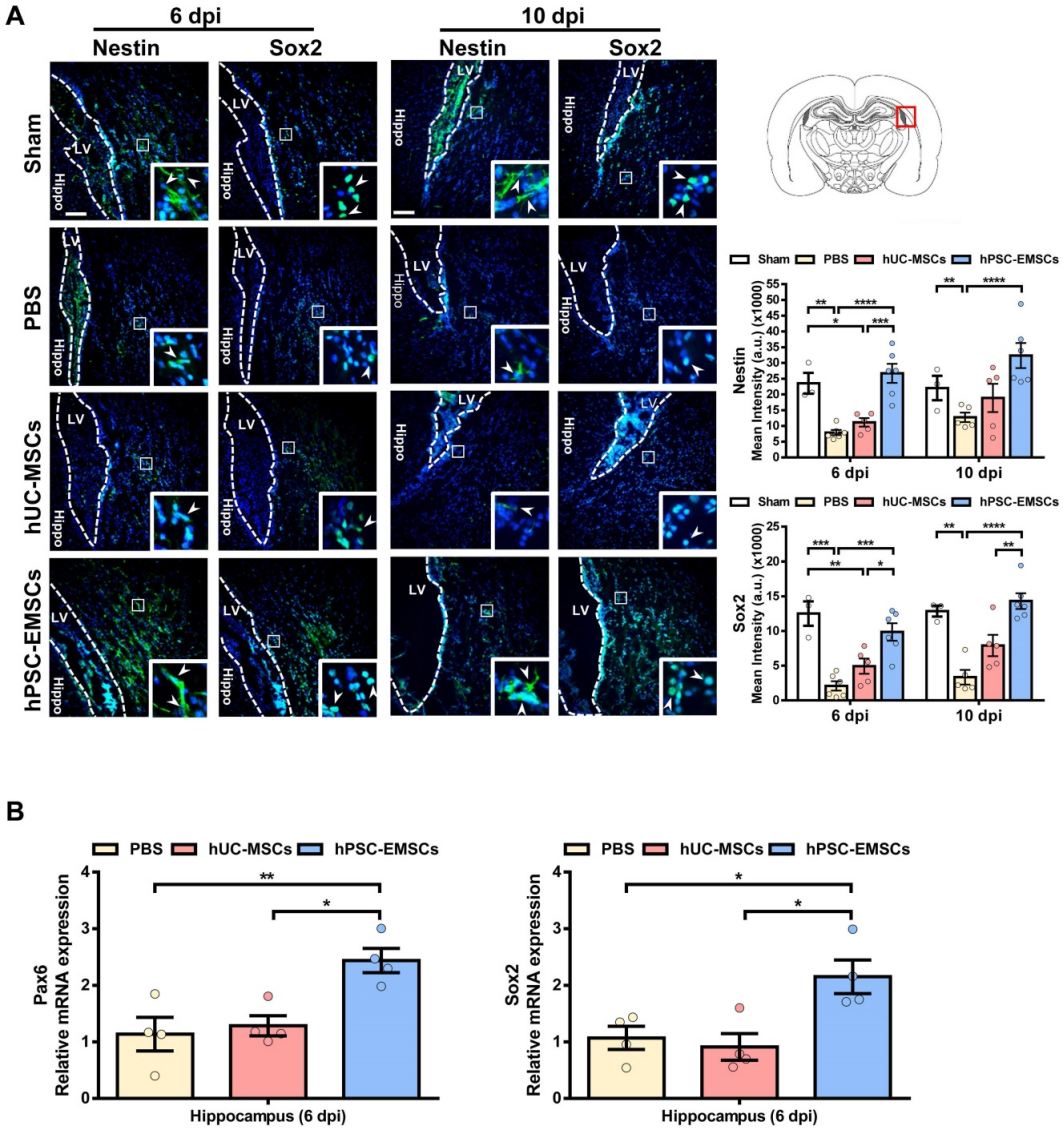
** hPSC-EMSCs increase endogenous NPC population and promote neurogenesis in HIE brain more effectively than hUC-MSCs.** The third day after HI (PN 10), 2 × 10^5^ hUC-MSCs or hPSC-EMSCs (derived from H7 or iPSCs) in 5 µL of PBS or vehicle were infused into the ipsilateral hemisphere. **A)** Representative immunofluorescent staining images of nestin and Sox2 in HIE rats in SVZ. Focused area in the SVZ is shown with a magnified view (inset) demonstrating nestin or Sox2 positive cells (green, arrowhead). The intensity of nestin and Sox2 was evaluated in 3-4 consecutive section (6 dpi, bregma -1.7 mm; 10 dpi, bregma -2.0 mm) and within 450 µm from the SVZ to indicate predominant newborn cells, which had migrated from the SVZ, scale bar: 100 µm. Quantification data represent mean ± SEM (6 dpi, sham: n = 3, PBS: n = 6, hUC-MSCs: n = 5, hPSC-EMSCs: n = 6; 10 dpi, sham: n = 3, PBS: n = 5, hUC-MSCs: n = 5, hPSC-EMSCs: n = 6). *, **, ***, **** represent *p* < 0.05, 0.01, 0.001 and 0.0001 respectively by Tukey's *post-hoc* test when statistical significance by One-way ANOVA (*p* < 0.05) was obtained; **B)** mRNA expression levels of *Pax6* and *Sox2* in HIE brain tissue collected from PBS-, hUC-MSCs- and hPSC-EMSCs-treated rats on 6 dpi. hPSC-EMSC transplantation significantly increased the expression of neural progenitor gene (*Sox2*) and neuron precursor gene (*Pax6*) in the hippocampal region more effectively than hUC-MSCs. Quantification data represent mean ± SEM, n = 4. *, **, ***, **** represent p < 0.05, 0.01, 0.001 and 0.0001 respectively by Tukey's *post-hoc* test when statistical significance by One-way ANOVA (*p* < 0.05) was obtained.

To further explore the mechanisms underlying the greater effects of hPSC-EMSCs, we stained HIE brain sections with the NPC markers, nestin and Sox2. We observed that hPSC-EMSCs were more effective than hUC-MSCs at inducing the NPC population in the SVZ in HIE brains (Figure 2A). In the sham rats, we observed a significant number of nestin^+^ or Sox2^+^ cells migrating from the SVZ at PN16-20; however, this phenomenon was markedly suppressed by HI insult. While hUC-MSCs did not show a significant effect, hPSC-EMSC treatment dramatically increased the number of NPCs in the SVZ region (Figure 2A). Consistent with the immunofluorescence data, RT-qPCR analysis of NPC markers in the hippocampal region showed the upregulation of *Pax6* and *Sox2* expression levels in the hPSC-EMSC-transplanted HIE brains compared with PBS- or hUC-MSC-transplanted brains (Figure 2B). In addition, while the mRNA expression levels of *nestin* and *Pax6* showed no differences in the neocortex after stem cell treatment (Figure S4D), the expression level of the immature neuronal marker, *Tubb3*, was mildly increased in the cortex after hPSC-EMSC treatment (Figure S4D). As the hippocampus and SVZ are the main regions of adult neurogenesis, these results indicate that hPSC-EMSCs promote neurogenesis via the induction of endogenous NPCs after postnatal brain injury.

**Figure 3:**
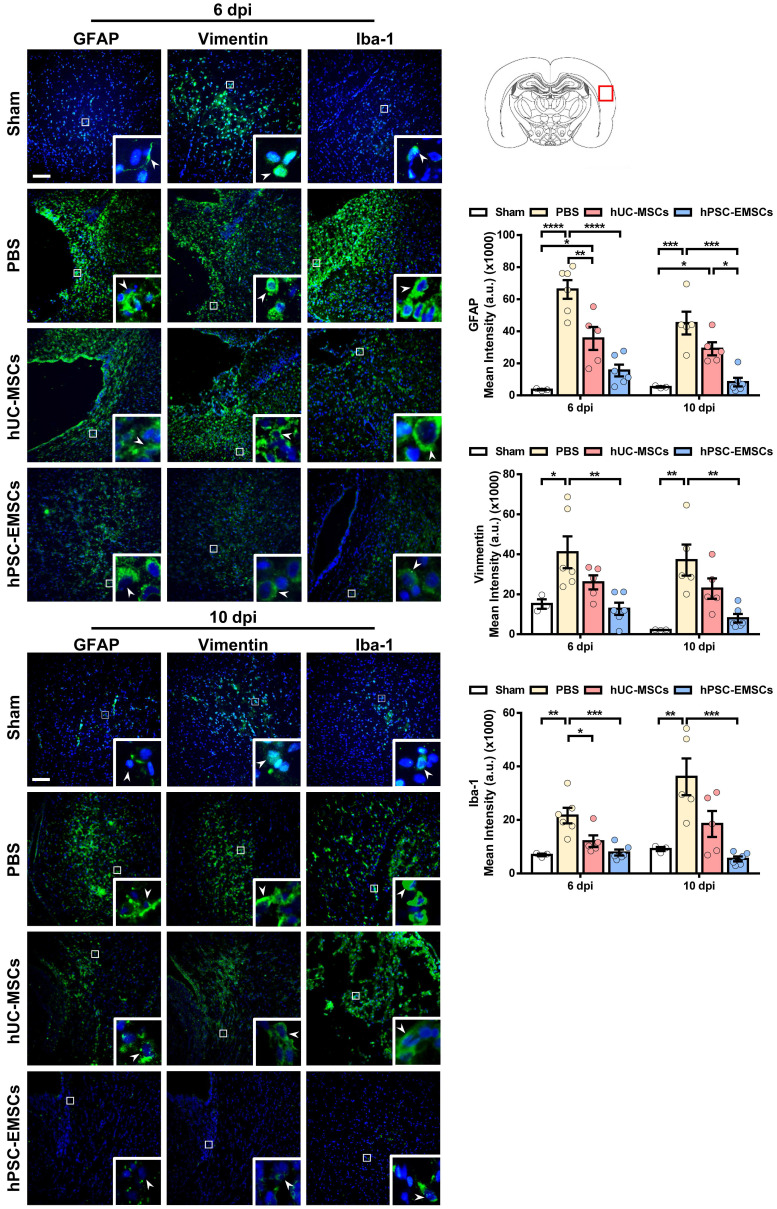
** hPSC-EMSCs dampen the activation of glial and microglial cells more effectively than hUC-MSCs in HIE rats.** The third day after HI (PN 10), 2 × 10^5^ hUC-MSCs or hPSC-EMSCs (derived from H7 or iPSCs) in 5 μL of PBS or vehicle were infused into the ipsilateral hemisphere. **A)** Representative immunofluorescent staining images of GFAP-, vimentin- and Iba-1-stained HIE rats in the cortex. Focused area in the brain cortex is shown with a magnified view(inset) demonstrating GFAP or vimentin or Iba-1 positive cells (green, arrowhead). The average intensity of GFAP, vimentin and Iba-1 was measured in three coronal sections (6 dpi, bregma -1.7, -1.8 and -1.9 mm; 10 dpi, bregma -2.0, -2.1 and -2.2 mm), scale bar: 100 µm. Quantification data represent mean ± SEM (6 dpi, sham: n = 3, PBS: n = 6, hUC-MSCs: n = 5, hPSC-EMSCs: n = 6; 10 dpi, sham: n = 3, PBS: n = 5, hUC-MSCs: n = 5, hPSC-EMSCs: n = 6). *, **, ***, **** represent *p* < 0.05, 0.01, 0.001 and 0.0001 respectively by Tukey's *post-hoc* test when statistical significance by One-way ANOVA (*p* < 0.05) was obtained.

Activation of the inflammatory cascade in HIE is characterised by the rapid induction of resident microglial and/or astroglial cells following HI insult. Once activated, these cells contribute to neuronal damage through the release of cytotoxic mediators 42. To evaluate whether MSC transplantation results in the inhibition of astrogliosis and microgliosis, MSC-transplanted brain sections were stained for the activated astrocyte markers, glial fibrillary acidic protein (GFAP) and vimentin, and for the activated microglial marker, Iba-1. While hUC-MSCs showed a suppressive effect on GFAP expression at 6 dpi, hPSC-EMSCs significantly reduced both GFAP and vimentin expression levels at 6 dpi and 10 dpi, indicating that hPSC-EMSCs had a stronger inhibitory effect on astroglial activation (Figure 3). Similarly, while hUC-MSCs reduced Iba-1 expression levels only at 6 dpi, a more substantial reduction was detected in the hPSC-EMSC-treated rats than in the hUC-MSC-treated rats (Figure 3). Altogether, these results indicate that hPSC-EMSCs promote a greater repair response than hUC-MSCs after HIE. This effect is likely achieved by promoting neurogenesis and mitigating astrogliosis and microgliosis.

### The recovery of motor and cognitive function is promoted to a greater extent by hPSC-EMSCs than hUC-MSCs in rats with HIE

**Figure 4:**
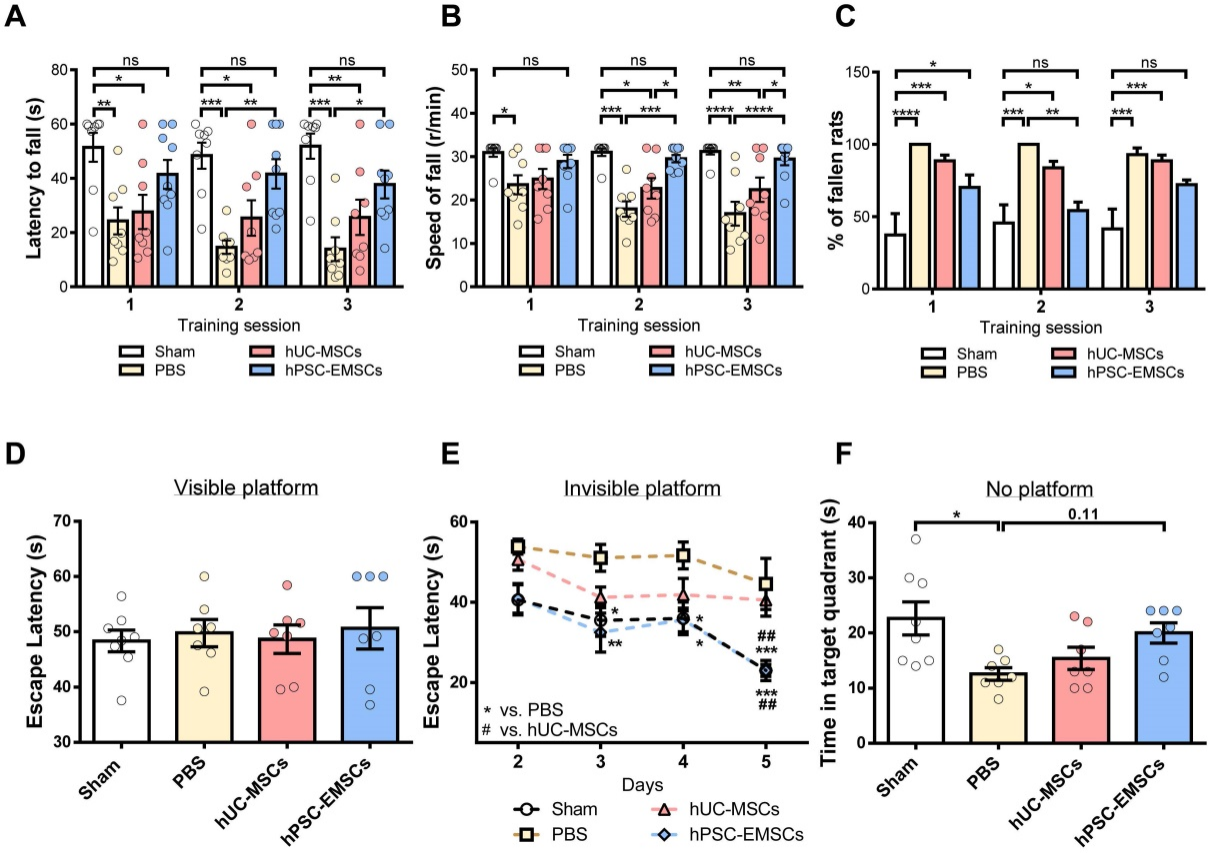
** hPSC-EMSC restore motor and learning functions better than hUC-MSCs in HIE rats.** The third day after HI (PN 10), 2 × 10^5^ hUC-MSCs or hPSC-EMSCs (derived from iPSCs) in 5 μL of PBS or vehicle were infused into the ipsilateral hemisphere. **A-C)** Assessment of motor and balancing function by rotarod test. Sham: n = 8, PBS: n = 8, hUC-MSCs: n = 8, hPSC-EMSCs: n = 9; **A-B)** hPSC-EMSC-treated rats displayed the highest latency to fall A) and required significantly higher speed to fall from the rotarod B) in comparison to PBS- and hUC-MSC- treated rats at training sessions 2 (S2) and 3 (S3); **C)** Percentage of hPSC-EMSC-treated rats falling from rotarod was significantly lower than PBS- and hUC-MSC-treated rats at training session 2 and 3. *, **, *** and **** represent *p* < 0.05, 0.01, 0.001 and 0.0001 respectively by Tukey's *post-hoc* test when statistical significance by One-way ANOVA (*p* < 0.05) was obtained; **D-F)** Assessment of learning and memory function by Morris water maze. sham: n = 8, PBS: n = 7, hUC-MSCs: n = 7, hPSC-EMSCs: n = 7; **D)** No difference in escape latency was observed for all rat groups during visible platform conditioning trial; **E)** hPSC-EMSC- transplanted rats performed significantly better in learning than PBS- and hUC-MSC- transplanted rats, which was comparable to the sham rats during invisible platform learning trials. *, **, *** represent *p* < 0.05, 0.01 and 0.001 respectively compared to PBS group by Tukey's *post-hoc* test when statistical significance by Two-way ANOVA (*p* < 0.05) was obtained. ^##^ represents p<0.01 compared to hUC-MSCs group by Tukey's *post-hoc* test when statistical significance by Two-way ANOVA (*p* < 0.05) was obtained; **F)** No significant difference in memory performance among the PBS- hUC-MSC- and hPSC-EMSC-treated groups was observed during the memory test trial. * represents p < 0.05 by Tukey's *post-hoc* test when statistical significance by One-way ANOVA (*p* < 0.05) was obtained.

To determine whether hPSC-EMSCs have a greater therapeutic effect than hUC-MSCs on brain functional recovery in HIE rats, the motor and cognitive functions of rats with HIE were assessed using the rotarod test and the MWM, respectively. In the rotarod test, at PN21-23, the hUC-MSC-treated rats did not show any improvement; however, the hPSC-EMSC-transplanted rats consistently performed better than the PBS- and hUC-MSC-treated rats during the 2^nd^ or 3^rd^ sessions of training (Figure 4A-C). In particular, the hPSC-EMSC-transplanted rats were able to stay on the rotarod longer than the PBS-treated rats and did not perform significantly differently from the sham group in this task (Figure 4A). When assessing the rotational speed of the rotarod at which the rats fell, the hPSC-EMSC-transplanted rats demonstrated significantly better balancing and coordinated motor function than either the hUC-MSC-transplanted or PBS-treated rats during the 2^nd^ and 3^rd^ training sessions (Figure 4B). In the MWM assessment performed at approximately 2 weeks after stem cell treatment, the hUC-MSC- and hPSC-EMSC-transplanted rats performed significantly better than the PBS-treated rats in terms of their ability to escape onto the invisible platform. The stem cell-treated rats had lower escape latencies on days 3 and 4 of the learning process (Figure 4D, E). Notably, on day 5 of the learning process, the hPSC-EMSC-transplanted rats performed significantly better than the hUC-MSC-transplanted and PBS-treated groups. In addition, the escape latency of the hPSC-EMSC-treated rats was similar to that of the sham rats, indicating that hPSC-EMSC treatment almost completely recovered the learning ability of the rats with HIE (Figure 4E). However, when assessing the memory retention of the rats, the hPSC-EMSC-treated rats spent longer searching for the platform in the target quadrant than the hUC-MSC- and PBS-treated groups, although there was no significant difference in performance among the three groups (Figure 4F). Taken together, these results indicate that hPSC-EMSCs are more effective than hUC-MSCs at promoting brain functional recovery after HIE, especially certain aspects of neurological function, such as balancing and learning abilities.

### CM from hPSC-EMSCs demonstrates greater neuroprotective and neurogenesis-promoting effects than CM from hUC-MSCs *in vitro*

**Figure 5:**
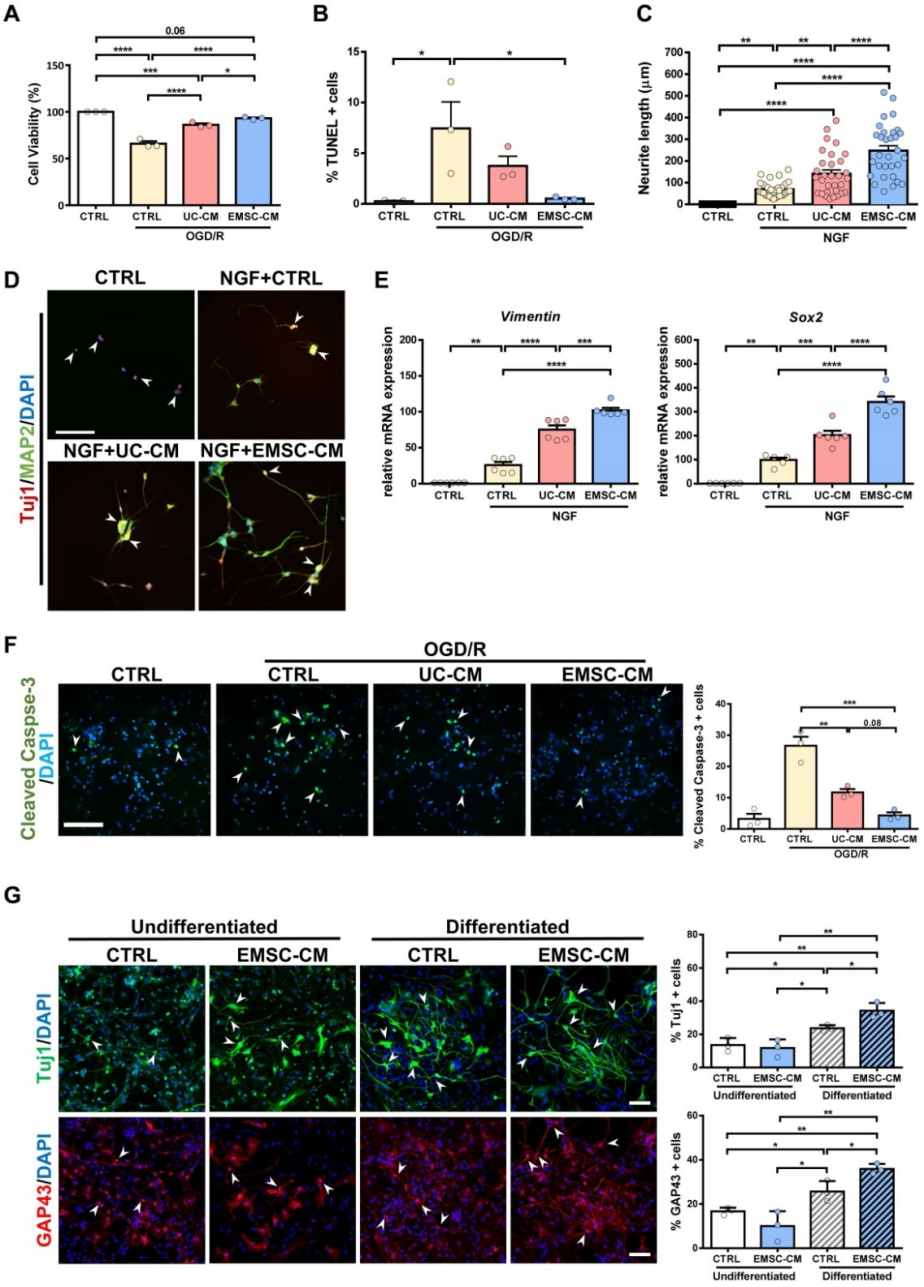
** CM from hPSC-EMSCs exhibits enhanced neuroprotective and neuritogenesis-promoting effects than that from hUC-MSCs. A)** PC-12 cells were challenged with OGD/R (4 h OGD + 24 h re-oxygenation) with or without CM derived from either hPSC-EMSCs (iPSCs) or hUC-MSCs. MTS assay was used to determine cell viability, data shown is derived from three independent experiments. **B)** PC-12 cells were challenged with OGD/R (4 h OGD + 24 h re-oxygenation) with or without CM derived from either hPSC-EMSCs or hUC-MSCs. TUNEL assay was used to determine cellular apoptosis. Data shown is derived from three independent experiments. Five fields were analyzed per slide under the microscope and no less than 200 cells were analyzed for quantification data. **C)** Concurrent treatment of PC-12 cells with NGF (50 ng/mL) and UC-CM/ EMSC-CM (6 days) significantly improved neurite outgrowth in comparison to untreated control- and NGF-treated groups. In the presence of NGF, EMSC-CM-treated group has significantly longer neurite outgrowth than UC-CM-treated group. Data shown is derived from three independent experiments. **D)** Representative immunofluorescent staining images showed longer and more neurites in EMSC-CM+NGF treatment group compared to NGF alone or UC-CM+NGF group as labelled by Tuj1 and MAP2, scale bar: 100 µm. In control group, PC-12 cells only express immature neuronal marker Tuj1 (red, arrowhead), whereas cells in other groups express both Tuj1 and MAP2 (yellow, arrow). **E)** mRNA expression levels of *vimentin* and *Sox2* in PC-12 cells treated with NGF only, NGF+UC-CM or NGF+EMSC-CM in PC-12 cells for 6 days, treated group. Data shown is derived from two independent experiments with triplicates.** F)** Primary cortical neurons were exposed to OGD/R (4 h OGD + 24 h re-oxygenation) with or without CM derived from either hUC-MSCs or hPSC-EMSCs. OGD/R increased apoptotic cells in primary cortical neuron which was significantly reduced by EMSC-CM and UC-CM treatment, scale bar: 100 µm. Data shown is derived from three independent experiments. Five fields were analyzed per slide under the microscope and no less than 200 cells were analyzed for quantification data. Arrowheads indicate cleaved caspase-3 positive cells. **G)** Representative immunofluorescent staining of Tuj1 and GAP43. Concurrent treatment of rNPCs with neuron differentiation medium and EMSC-CM significantly increased Tuj1 and GAP43 positive cell populations in comparison to rNPCs treated with neuron differentiation medium only, scale bar: 100 µm. Data shown is derived from three independent experiments. Five fields were analyzed per slide under the microscope and no less than 200 cells were analyzed for quantification data. Arrowheads indicate Tuj1 or GAP43 positive cells. Data represent mean ± SEM, *, **, *** and **** represent *p* < 0.05, 0.01, 0.001 and 0.0001 respectively by Tukey's *post-hoc* test when statistical significance by One-way ANOVA (*p* < 0.05) was obtained.

As transplanted human MSCs have limited survivability and do not differentiate into neurons or glia in the brains of rats with HIE (Figure S3A, B), it is plausible that the therapeutic function of MSCs is associated with the promotion of endogenous repair through paracrine/trophic effects. To evaluate whether secretory factors from hUC-MSCs or hPSC-EMSCs elicit a neuroprotective effect, we used an OGD/R model on rat PC-12 cells to mimic HI insult and assessed the potential role of stem cell-derived CM *in vitro*. The PC-12 cells were challenged with OGD for 4 h and their survival rates were then monitored during the reperfusion phase, in the presence or absence of CM. We observed that the viability of both sets of CM-treated PC-12 cells was significantly increased compared with the OGD/R group without CM. However, the CM derived from hPSC-EMSCs showed a stronger effect on cell viability (Figure 5A). The presence of CM also caused a dramatic decrease in the number of apoptotic cells, with the CM derived from hPSC-EMSCs being more effective, thus substantiating the finding that hPSC-EMSCs have a superior neuroprotective effect (Figure 5B, S5A). In the presence of NGF, PC-12 cells differentiate into sympathetic neuron-like cells and are capable of substantial neurite outgrowth 43. These properties allow for the exploration of the possible effect of factors secreted by stem cells on the morphological differentiation of neurons. Indeed, the CM from both hUC-MSCs and hPSC-EMSCs promoted neurite outgrowth in the PC-12 cells in the presence of NGF, but significantly greater neurite outgrowth (*p* < 0.0001) was observed in the cells treated with CM derived from hPSC-EMSCs, which showed the greatest effect (Figure 5C). Prominent neurite outgrowth was further demonstrated by microtubule-associated protein 2 (MAP2) and neuron-specific class III beta-tubulin (Tuj1) staining, which showed the formation of longer and a greater number of neurites in the EMSC-CM-treated group (Figure 5D). In addition, RT-qPCR results indicated that the expression levels of neuronal genes, such as *Sox2* and vimentin, were significantly upregulated under NGF induction and further upregulated in the presence of CM, with a stronger effect observed in the EMSC-CM-treated group (Figure 5E).

To validate the cell line results, primary cultures of rat cortical neurons were exposed to OGD/R, in the presence or absence of CM derived from either hUC-MSCs or hPSC-EMSCs. Consistent with the cell line data, incubation with CM dramatically reduced the number of apoptotic cells after OGD/R challenge, with fewer apoptotic cells observed in the EMSC-CM-treated group than in the control group (Figure 5F). As endogenous neurogenesis was observed in the hippocampus and SVZ after hPSC-EMSC transplantation (Figure 2A, B), we isolated rNPCs to determine whether factors secreted by hPSC-EMSCs could promote neuronal differentiation *in vitro*. After 12 days of differentiation (Figure S5B), gene expression analysis of rNPCs in the presence of CM derived from hPSC-EMSCs showed a dramatic upregulation in the levels of the NPC and neuroblast markers, *Sox2* and *Dcx,* as well as the neuronal markers, *Nf200* and growth-associated protein* 43 (Gap43*)*,* indicating rNPC expansion and differentiation (Figure S5C). The differentiation capacity of rNPCs was further confirmed by immunocytochemical analysis of Tuj1 and GAP43, which are markers of developing and immature neurons. Higher levels of differentiation into a neuronal phenotype were observed in the CM-treated rNPCs, as the CM from hPSC-EMSCs was able to potentiate the neuronal differentiation of rNPCs (Figure 5G). Cumulatively, these results indicate that factors secreted by hPSC-EMSCs demonstrate high potential for neuroprotection and the promotion of neurogenesis.

### The gene expression signature of hPSC-EMSCs indicates active neuromodulatory potential through secreted factors

**Figure 6:**
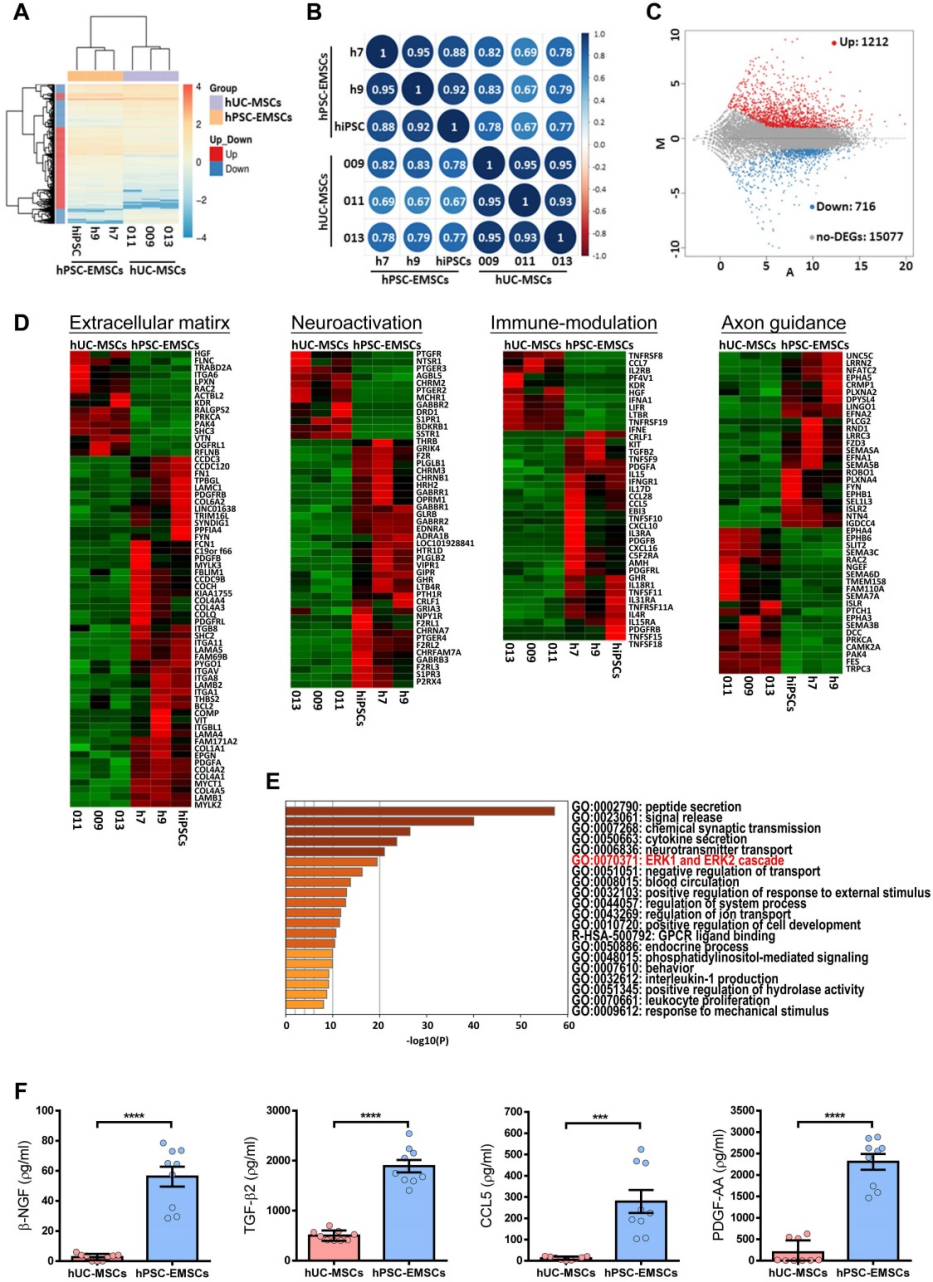
** hUC-MSCs and hPSC-EMSCs exhibit different gene expression profile by RNA-seq analysis. A)** Global gene expression profile analysis (hPSC-EMSCs (n = 3) Vs. hUC-MSCs (n = 3)) by RNA-seq indicated that hUC-MSCs and hPSC-EMSCs had different gene expression profiles from each other; **B)** Pearson's correlation analysis of hUC-MSCs and hPSC-EMSCs indicated that EMSCs derived from hPSCs was closely related to the EMSCs derived from hESCs and were distinct from hUC-MSCs. The correlation plot was generated using an online platform (http://www.bioinformatics.com.cn/); **C)** Comparison between hUC-MSCs and hPSC-EMSCs by MA plot indicated that 1212 genes were differentially upregulated and 716 genes were differentially downregulated in hPSC-EMSCs compared to hUC-MSCs (DEGs, Log_2_ Fold Change ≤ -1 or ≥ 1, P adj ≤ 0.05 or > 0.05); **D)** Heatmap analysis showing the differentially expressed genes associated with ECM, immunomodulation, neuroactivation and axon guidance between hUC-MSCs and hPSC-EMSCs; **E)** Gene ontology analysis of secretory factors (GO: 0032940) indicated that genes associated with ERK1 and ERK2 cascade were differentially expressed in hUC-MSCs and hPSC-EMSCs; **F)** ELISA analysis of β-NGF, TGF-β_2_, PDGF-AA and CCL5 in the CM derived from hPSC-EMSCs and hUC-MSCs. Three different EMSC-CM (from H7, H9 and iPSCs) and three UC-CM (from 009, 011 and 013) were used for the analysis. *** and **** represent *p* < 0.001 and 0.0001 respectively by Student T-test.

To understand the molecular mechanisms underlying the therapeutic effects of hPSC-EMSCs, the genes differentially expressed between hPSC-EMSCs and hUC-MSCs were determined using RNA-seq analysis. As shown in Figures 6A and S6A, each cell type clustered into separate groups, indicating that functionally distinct stem cell populations were obtained. Hierarchical clustering and Pearson's correlation analysis (Figure 6A, B) showed that EMSCs derived from iPSCs were more closely related to EMSCs derived from hESCs, all of which were distinct from hUC-MSCs. An MA plot analysis demonstrated that 1,928 genes were differentially expressed between hPSC-EMSCs and hUC-MSCs. Of these, 1,212 genes were upregulated and 716 genes were downregulated (log_2_ fold change > 1, P_adj_ < 0.05, Figure 6C). Gene ontology analysis revealed that the predominant differences between hPSC-EMSCs and hUC-MSCs were functional categories pertaining to extracellular matrix organisation, immunomodulation, neuroactive ligand-receptor interactions, axon guidance and synapse organisation (Figure 6D, S6B). The 200 most highly expressed genes in hPSC-EMSCs are listed in Table S3. Of note, multiple NC genes, including *Gbx2*,* Dlx1*,* Zic1*,* Tfap2c* and *Lhx1,* were expressed only by hPSC-EMSCs, thus confirming their EMSC identity. In addition, hPSC-EMSCs showed high expression levels of a panel of genes that were not expressed by hUC-MSCs, such as *Sema5b*,* Vit*,* Adcyap1*,* Pdgf-bb*,* Btc*,* Ache* and *Ntn4* (Figure S6C and Table S3). To further investigate the potential link between the therapeutic effects of MSCs and their paracrine factors, we focused on the secreted factors (n = 93 transcripts) upregulated in hPSC-EMSCs compared with hUC-MSCs. Most genes on this list are involved in peptide secretion, synaptic transmission, neurotransmitter transport and cytokine secretion (Figure 6E, S6C; Table S4). For instance, Ngf, Pdgf-aa, Adap12, ApoE and Adcyap1 have been shown to promote neurogenesis and axonal outgrowth via multifaceted mechanisms following traumatic brain injury 44-47. Alternatively, Ccl5, Ptgs1, Tnfsf11 and Tnfsf15 are strong regulators of the inflammatory response and demonstrate immunomodulatory effects after brain damage 48-50. The enrichment of several secretory factors in hPSC-EMSCs was confirmed using ELISAs (Figure 6F, S6D).

### hPSC-EMSCs promote neuroregeneration by activating the ERK/CREB signalling pathway

**Figure 7:**
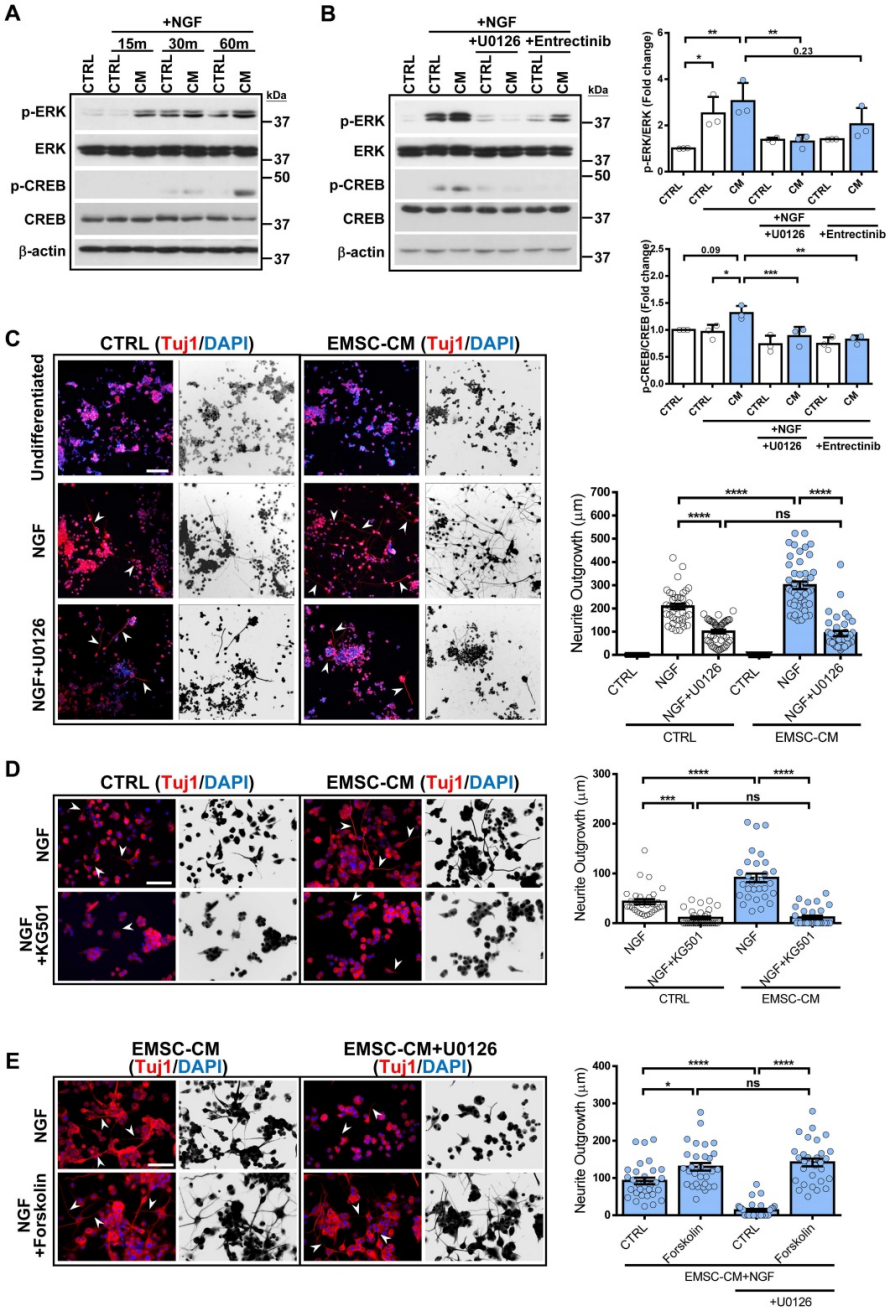
** CM from hPSC-EMSCs promotes neurogenesis and neuritogensis via the ERK/CREB signaling pathway. A)** PC-12 cells were treated with NGF for indicated different time points with or without the CM derived from hPSC-EMSCs (iPSCs). Representative images of western blotting showed that the expression levels of p**-**ERK and p-CREB were higher in EMSC-CM-treated group compared to control group at 60 min; **B)** Representative blot image and quantification of western blotting result showed that CM-potentiated increase of p-ERK and p-CREB was alleviated by U0126 and Entrectinib (10 μM U0126, 10 nM Entrectinib); **C)** PC-12 cells exhibited enhanced neurite outgrowth and neurite length by Tuj1 staining when treated with NGF and EMSC-CM concurrently, and were remarkably reduced in the presence of U0126 (10μM). Arrowheads indicate PC-12 cells with neurites. Images were presented as the reverse grayscale (black on white) as well, scale bar: 100 µm; **D)** PC-12 cells exhibited enhanced neurite outgrowth and neurite length by Tuj1 staining when treated with NGF and EMSC-CM concurrently, and were remarkably reduced in the presence of KG501(1μM). Arrowheads indicate the cells with neurites. Images were presented as the reverse grayscale (black on white) as well, scale bar: 100 µm; **E)** Forskolin (10μM) promoted neuritogenesis of PC-12, the effects could not be reversed by U0126, scale bar=100μm. Arrowheads indicate PC-12 cells with neurites. Experiments were repeated at least three times, and quantification data represent mean ± SEM, *, **, *** and **** represent *p* < 0.05, 0.01, 0.001, and 0.0001 by Tukey's *post-hoc* test when statistical significance by One-way ANOVA (*p* < 0.05) was obtained.

While attempting to identify the link between molecules secreted by hPSC-EMSCs and their neuromodulatory effects, we noticed that the ERK1/2 signalling pathway was the most significantly different signalling pathway associated with the secreted factors enriched in hPSC-EMSCs compared with hUC-MSCs (Figure 6E). We used the Metascape bioinformatics tool to identify protein-protein interaction-based networks focused on ERK1/2 activation (Figure S6E). Given that robust neuritogenesis and neurogenesis were observed after hPSC-EMSC treatment both *in vitro* and *in vivo*, we speculated that activation of the ERK1/2 pathway may underlie the positive effects of hPSC-EMSCs on neuroregeneration. We first used an NGF-induced PC-12 neurogenesis model and found that NGF evoked rapid activation of ERK, as indicated by the upregulation of phospho (p)-ERK levels, which peaked at 30 min. Importantly, the CM derived from hPSC-EMSCs potentiated the NGF-induced increase in p-ERK levels (Figure 7A). It is well-established that the activation of CREB initiates the transcription of genes associated with neuritogenesis and neurogenesis 51, and that the ERK pathway is an upstream regulator of CREB. In accordance with this, factors secreted from hPSC-EMSCs potentiated the NGF-induced activation of CREB, as shown by the upregulation of p-CREB levels (Figure 7A). Meanwhile, blocking ERK activation with U0126 completely abolished the CM-potentiated, NGF-induced increase in p-ERK and p-CREB levels. Notably, blocking the NGF receptor, TrkA, with entrectinib partially reduced the levels of p-ERK (Figure 7B), indicating that other secretory molecules in the CM, besides NGF, are able to activate the ERK/CREB pathway. Moreover, U0126 and the CREB inhibitor, KG501, completely abolished CM-potentiated neuritogenesis in PC-12 cells (Figure 7C and D). In contrast, activation of CREB by forskolin promoted neuritogenesis. This effect was not alleviated by U0126, indicating that CREB is downstream of ERK (Figure 7E). Next, we examined the activation of ERK in rNPCs after neuronal differentiation in the presence or absence of CM. Both cytoplasmic and nuclear p-ERK levels were significantly upregulated in the CM-treated rNPCs after differentiation (Figure S7A), supporting the central role of ERK activation in the stimulatory effects of hPSC-EMSCs on neurogenesis. Additionally, the suppression of ERK or CREB activity significantly alleviated the potentiation effect of EMSC-CM on neuronal differentiation (Figure S7B). Collectively, these results provide strong evidence that the ERK/CREB pathway mediates the positive effects of hPSC-EMSCs on neuroregeneration.

### Intranasally administered CM derived from hPSC-EMSCs promotes brain injury repair in rats with HIE

**Figure 8:**
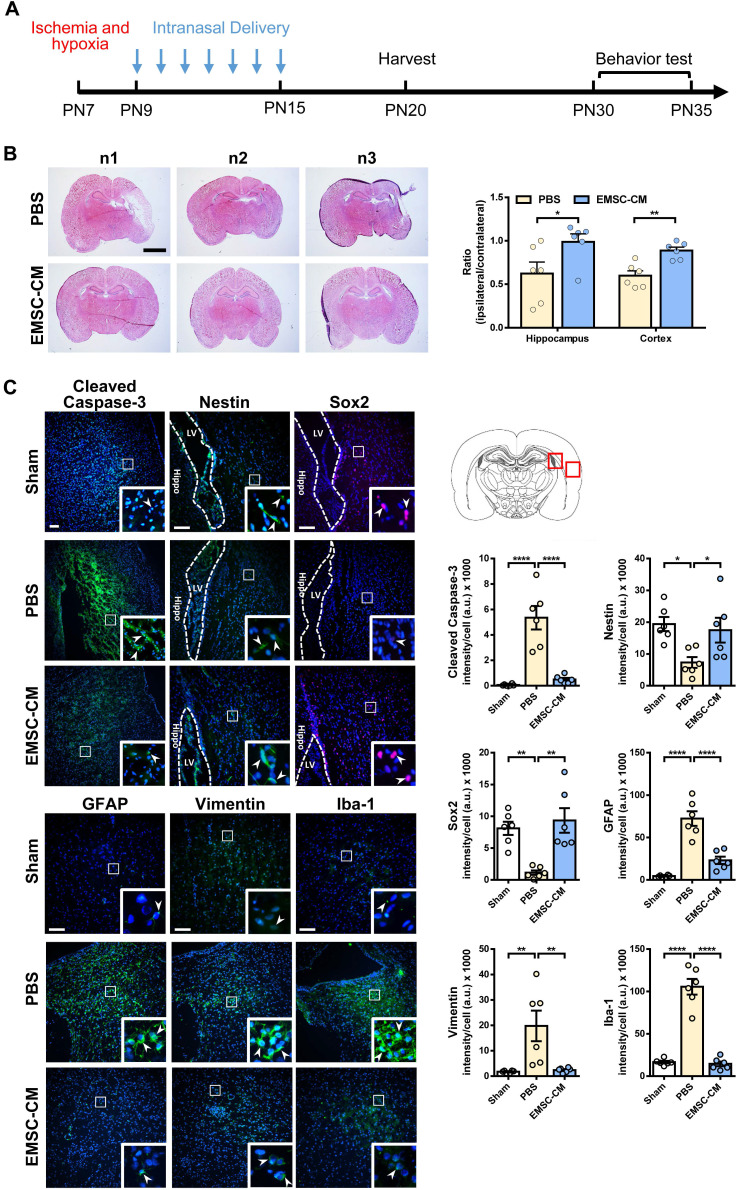
** Intranasal delivery of the CM derived from hPSC-EMSCs promotes brain injury repair in HIE rats. A)** Scheme presentation of CM (derived from iPSCs) administration in HIE rats; **B)** Bright field images of H&E stained ipsilateral/ contralateral brain cryosections collected from PBS- and EMSC-CM-treated rats, n = 6, scale bar: 2.5 mm. *, ** represent *p* < 0.05 and 0.01 respectively by Tukey's *post-hoc* test when statistical significance by One-way ANOVA (*p* < 0.05) was obtained; **C)** Representative immunofluorescent staining images of cleaved caspase-3, nestin, Sox2, GFAP, vimentin and Iba-1 in HIE rats in the cortex and SVZ. Focused area in the SVZ and cortex is shown with a magnified view(inset) demonstrating positive cells (arrowhead). The average intensity of cleaved caspase-3, GFAP, vimentin and Iba-1 was measured in three coronal sections (bregma -2.0, -2.1 and -2.2 mm), The intensity of nestin and Sox2 were evaluated in three consecutive section (bregma -2.0 mm) and within 450 µm from the SVZ to indicate predominant newborn cells, which had migrated from the SVZ, scale bar: 100 µm. Quantification data represent mean ± SEM, (sham: n = 6, PBS: n = 6, EMSC-CM: n = 6). *, ** and **** represent *p* < 0.05, 0.01 and 0.0001 respectively by Tukey's *post-hoc* test when statistical significance by One-way ANOVA (*p* < 0.05) was obtained.

**Figure 9:**
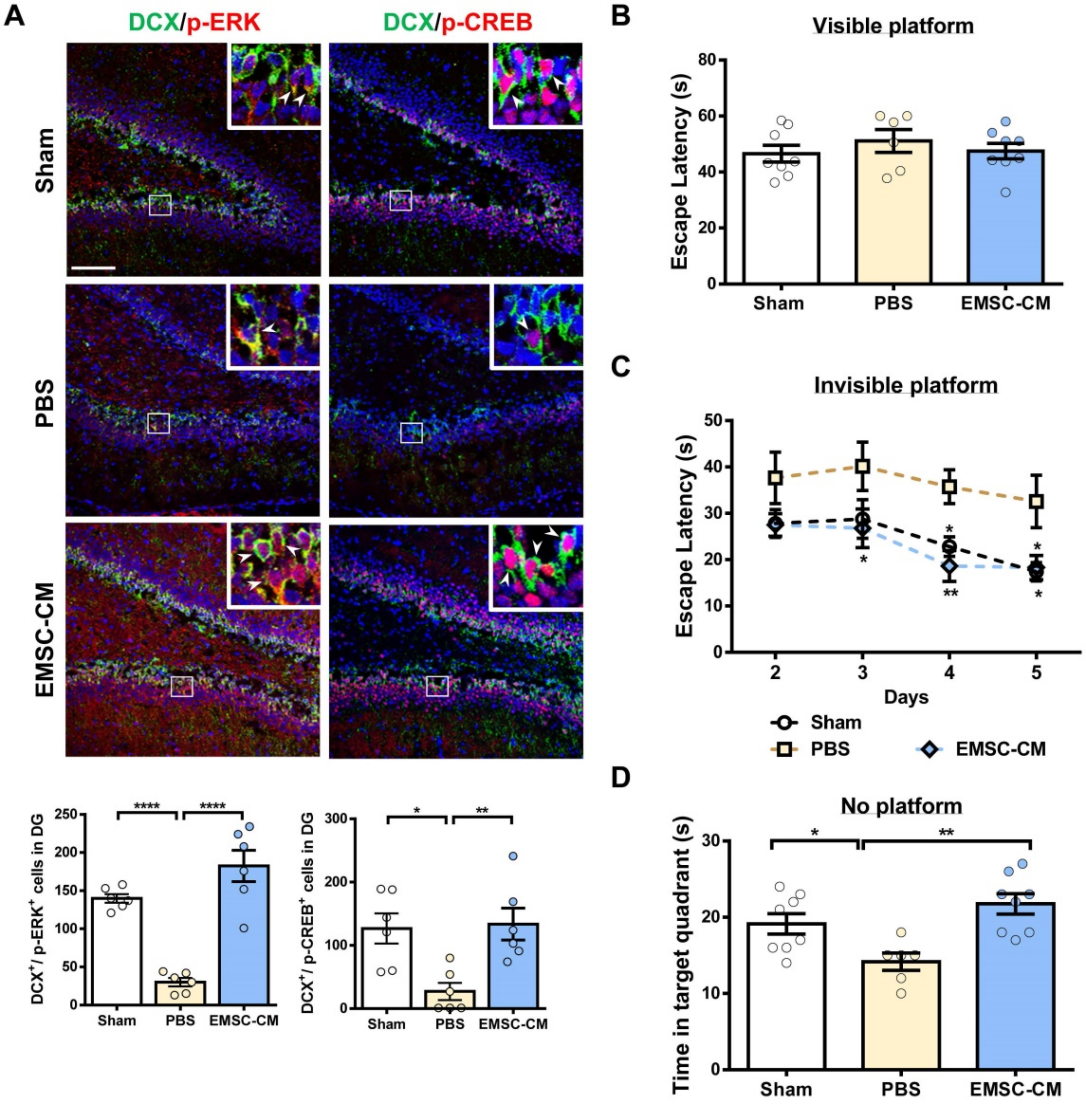
** Intranasal delivery of the CM derived from hPSC-EMSCs promotes brain functional recovery in HIE rats. A)** Representative immunofluorescent staining images of DCX, p-ERK and p-CREB in HIE rats in the dentate gyrus (DG). Focused area in the DG is shown with a magnified view (inset) demonstrating DCX/p-ERK or DCX/p-CREB double positive cells (arrowhead). The average intensity was measured in three consecutive coronal sections (bregma -2.0 mm), scale bar: 100 µm. Quantification data represent mean ± SEM, (sham: n = 6, PBS: n = 6, EMSC-CM: n = 6). *, *** and **** represent *p* < 0.05, 0.001 and 0.0001 respectively by Tukey's *post-hoc* test when statistical significance by One-way ANOVA (*p* < 0.05) was obtained; **B-D)** Assessment of learning and memory function by Morris water maze. sham: n = 8, PBS: n = 6, EMSC-CM: n = 8; **B)** No difference in escape latency was observed for all rat groups during visible platform conditioning trial; **C)** EMSC-CM- transplanted rats performed significantly better in learning than PBS - transplanted rats, which was comparable to the normal rats during invisible platform learning trials. *, ** represent *p* < 0.05 and 0.01 respectively compared to PBS group by Tukey's *post-hoc* test when statistical significance by Two-way ANOVA (*p* <0.05) was obtained; **D)** EMSC-CM- transplanted rats performed significantly better in memory retention than PBS - transplanted rats. *, ** represent p< 0.05 and 0.01by Tukey's *post-hoc* test when statistical significance by One-way ANOVA (*p* < 0.05) was obtained.

Intranasal administration of stem cells and/or therapeutic agents has shown promise in preclinical and clinical settings because of its ability to bypass the blood-brain barrier 52, 53. To further validate the role of secretory factors in mediating the therapeutic effects of hPSC-EMSCs, we determined whether the intranasal administration of CM derived from hPSC-EMSCs could promote brain injury repair after HI. We observed that repetitive intranasal administration of secretory factors for 7 days (Figure 8A) dramatically reduced brain lesion size (Figure 8B). In particular, the size of the hippocampus was almost completely recovered on the ipsilateral side and was comparable to the contralateral side after CM treatment. Consistent with the cell treatment data, CM administration dramatically reduced the number of cells positive for cleaved caspase-3, GFAP, vimentin and Iba-1, but increased the number of cells positive for nestin and Sox2 in the SVZ region (Figure 8C). To further confirm that the ERK/CREB pathway underlies the therapeutic effects of hPSC-EMSCs, we examined the levels of p-ERK and p-CREB in the hippocampi of EMSC-CM-treated rats with HIE. As shown in Figure 9A, strong signals were observed in the dentate gyrus (DG) of the hippocampus in sham rats. However, the number of DCX^+^ cells and p-ERK^+^ or p-CREB^+^ cells were dramatically reduced upon HI-induced damage. EMSC-CM treatment completely restored the number of DCX^+^/p-ERK^+^ and DCX^+^/p-CREB^+^ cells in the DG of rats with HIE, indicating that the ERK/CREB axis mediates the neurogenesis-promoting effect of hPSC-EMSCs. Moreover, in the MWM test conducted at PN30-35, EMSC-CM-treated rats performed significantly better than PBS-treated rats and had lower escape latencies on day 3-5 of the learning process, showing no difference compared to the sham rats (Figure 9B, C). In addition, the EMSC-CM-treated rats demonstrated greater memory retention than the PBS-treated rats (Figure 9D). Altogether, these data clearly demonstrate that factors secreted by hPSC-EMSCs reduce lesion size, apoptosis and inflammatory responses, but promote endogenous neurogenesis, in rats with HIE.

## Discussion

Here, we report that hPSC-EMSCs are advantageous for HIE treatment due to their multifaceted neuromodulatory activities. To the best of our knowledge, this is the first report showing the beneficial effects of EMSCs derived from PSCs on brain damage repair. Moreover, we identified a panel of secreted factors that are highly enriched in hPSC-EMSCs and are able to activate the ERK/CREB axis, thus providing a mechanism underlying the enhanced therapeutic effects of hPSC-EMSCs.

The successful clinical application of adult MSCs is hampered by invasive isolation procedures, low yields and reduced stem cell potency after cultivation. As such, MSCs derived from extraembryonic tissues, such as the placenta, umbilical cord or amniotic fluid, are more favourable, as these cells have a greater expansion capacity and are considered biological waste by the body. In particular, hUC-MSCs have been recently tested for their efficacy in treating paediatric brain damage, with encouraging results 54-56. However, high variability in therapeutic efficacy has been noted due to the diversity and heterogeneity of isolated cells. Indeed, analyses of individual colonies and single-cell RNA-seq data clearly indicate that subpopulations of hUC-MSCs may have different biological properties and therapeutic functions 57, 58. Another critical limitation of hUC-MSC therapy is the lack of sufficient information regarding its mechanism of action. Given these limitations, PSC-derived MSCs are considered potential alternatives due to their high proliferation capacity, unlimited supply and relative homogeneity. Of note, although EMSCs derived from adult tissues, such as dental pulp or cranial bones, have been recently evaluated for their ability to treat neurological diseases 25, 59-62, the potential benefits of PSC-derived EMSCs have not been studied. In addition, EMSCs have not been compared to other adult MSCs for the quantity or quality of their therapeutic efficacy. In this study, we directly compared the therapeutic effects of hPSC-EMSCs with those of hUC-MSCs in the treatment of HIE. Our results clearly demonstrated that, while both hUC-MSCs and hPSC-EMSCs promoted brain damage repair, hPSC-EMSCs were more effective at ameliorating histological abnormalities and reducing brain infarction volume (Figure 1B). In concordance with these data, motor function and learning ability were more significantly improved in hPSC-EMSC-transplanted rats (Figure 4).

Further histological characterisation of the brain tissues revealed that engrafted hPSC-EMSCs exhibited greater effects than hUC-MSCs on (a) alleviating the apoptotic response, (b) promoting neuro-regeneration by inducing endogenous stem/progenitor cell activation and neurogenesis and (c) inhibiting injury-related inflammatory responses (Figure 2-3). Similar to other adult stem cells, hUC-MSCs have been shown to improve functional outcomes and increase the number of mature neurons and synaptic plasticity 63, 64. hUC-MSCs also significantly decrease brain damage in some cases linked to reduced apoptosis, astrogliosis and microglial activation 65, 66. Our results are consistent with the results of all of these studies showing the beneficial effects of hUC-MSCs on promoting histological and functional recovery after HI insult, albeit with milder effects compared to hPSC-MSCs. In addition, while hUC-MSCs did not show detectable effects on promoting endogenous neurogenesis, hPSC-MSCs dramatically increased the number of neural progenitor cells in the SVZ and hippocampus. These data indicate that hPSC-EMSCs are advantageous for treating HIE by changing the hostile, damaged environment to a regeneration/repair-friendly environment.

In this study, we also conducted the first molecular comparison of PSC-EMSCs and hUC-MSCs using RNA-seq analysis. hPSC-EMSCs expressed many genes associated with extracellular and cell-surface regions, immunomodulation, NC development, neuroactivation and synapse organisation at levels at least two-fold higher than in hUC-MSCs (Table S3). This indicates that hPSC-EMSCs belong to an ectomesenchymal stem cell-like population that is endowed with neural traits and closely interacts with other cells. Given that < 1% of hPSC-EMSCs were detected at 10 dpi after intracranial administration, we focused on the secretory factor-related genes that are implicated in neurogenesis, synaptic regulation and axon guidance (Figure 6E, S6C). We identified a panel of secretory factors that are unique to hPSC-EMSCs and are not expressed by hUC-MSCs, such as *Adcyap1*, *Btc*, *Ache*, *Ntn4*, *Vit*, *Sema5b* and* Pdgf-bb*. *Adcyap1* encodes a secreted proprotein that is further processed into multiple mature peptides to stimulate adenylate cyclase and increase cAMP levels, resulting in the transcriptional activation of the target genes. One of its peptide products, pituitary adenylate cyclase-activating polypeptide (PACAP) is a highly conserved neuropeptide that regulates neuronal physiology 67. Ntn4 is widely expressed in the brain and is involved in axon guidance and neuronal plasticity 68. Sema5b plays an important role in axon guidance and growth by triggering the dynamic rearrangement of the actin cytoskeleton in the neuronal growth cone 69. The differential expression profile of hUC-MSCs and hPSC-MSCs raises the question of whether neuromodulatory activity may also differ between the two cell types. Therefore, *in vitro* studies using CM derived from hUC-MSCs and hPSC-EMSCs were designed to evaluate the role of MSC secretory factors in mediating neuroprotection, neuritogenesis and neurogenesis. Consistent with the *in vivo* data, CM derived from hPSC-EMSCs induced a stronger neuroprotective effect, and promoted neurogenesis and neurite outgrowth to a significantly higher extent than CM derived from hUC-MSCs (Figure 5). These effects were further validated by experiments showing that direct administration of the CM derived from hPSC-EMSCs significantly improved the learning and memory abilities of rats with HIE (Figure 8, 9). Thus, it is clear that hPSC-EMSCs elicit a greater therapeutic effect through their unique paracrine effects on neuroprotection and neuroregeneration. Nevertheless, it should also be noted that serum deprivation has been shown to modify the energy-metabolic profile and cytokine production of MSCs 70, 71. Therefore, we cannot completely exclude the possibility that preconditioning with serum deprivation partially contributed to the observed beneficial effects of CM derived from hPSC-EMSCs.

Another notable finding from this study is that the secretory factors derived from hPSC-EMSCs promote neurogenesis via the ERK/CREB pathway. Sustained ERK activation and nuclear translocation, which can be achieved through several signalling pathways, including those mediated by phosphoinositide 3-kinase, Ras, Ca^2+^, protein kinase C and cAMP, are required for the induction of neurogenesis 72, 73. We noticed that, out of the 93 genes that were associated with MSC secretion, 21 were related to the ERK pathway (Figure 6E, S6E). Using ELISAs, we confirmed the dramatic upregulation of several secretory factors, including NGF, PDGF-AA, CCL5 and TGFβ_2_, in the CM derived from hPSC-EMSCs (Figure 6F). All of these factors can activate the ERK/CREB pathway and are implicated in neurogenesis, axon guidance and/or synaptic regulation. NGF is a neurotropic factor that has a well-established role in activating cellular signalling cascades, such as ERK/CREB, and in regulating neuronal proliferation, differentiation and survival. Alternatively, PDGF-AA is a strong activator of the ERK pathway and prevents oxidative stress-induced neuronal death 74. Importantly, we observed that the TrkA inhibitor, entrectinib, only partially reversed the CM-potentiated activation of ERK (Figure 7B), supporting the scenario that multiple secretory factors, aside from NGF, collaborate to activate the ERK/CREB pathway in the recipient neurons. ERK and CREB inhibitors completely abolished the CM-potentiated activation of neuritogenesis, indicating that the ERK/CREB pathway mediates the neuritogenesis-promoting effect of hPSC-EMSCs. In another model of neurogenesis using primary rNPCs, CM derived from hPSC-EMSCs evoked sustained ERK activation and its concomitant translocation to the nucleus, which is similar to the effects of neurogenic factors, such as NGF, PACAP and retinoic acid 75, 76. In addition, suppression of the ERK/CREB axis abolished the potentiation effect of hPSC-EMSCs on the neuronal differentiation of rNPCs. The results presented in this study suggest that sustained activation of the ERK/CREB axis is one of the mechanisms by which hPSC-EMSCs promote neurogenesis.

## Limitations and future directions

HI insult activates a series of time-dependent pathophysiological responses in the brain. While we observed that hPSC-EMSCs inhibited astrogliosis and microgliosis in rats with HIE, this study only focused on the neuromodulatory effects of hPSC-EMSCs. The regulatory role of hPSC-EMSCs in the inflammatory response at the early phase of HIE was not addressed. Consistent with the results of previous studies, sex differences were observed in HIE disease progression and the therapeutic effectiveness of the EMSCs. The mechanisms responsible for these differences require further investigation. We demonstrated that the intranasal delivery of secretory factors derived from hPSC-EMSCs was effective at promoting brain functional recovery after HI; however, the efficiency and stability of such a delivery method needs to be further evaluated in future studies.

## Conclusions

In summary, our findings suggest that hPSC-EMSCs are promising cells for HIE treatment. In terms of clinical application, hPSC-EMSCs present several advantages: 1) infinitely expandable hPSCs serve as a potentially unlimited source; 2) hPSC-MSCs are at a much earlier developmental stage than adult MSCs and are therefore more potent; 3) hPSC-MSCs are endowed with both neural and mesenchymal traits and 4) hPSC-MSCs secrete unique bioactive factors that promote neuroprotection and neuroregeneration and thus are more favourable for use in the treatment of ischaemic brain disease. However, it should be noted that the safety and effectiveness of MSC therapy is still debated, as the injection of cellular components may induce adverse effects, such as infection, immune responses and a potential risk of tumourigenicity. In the light of these restrictions, MSC-derived secretory factors offer an alternative strategy for promoting tissue repair. The findings that the factors secreted from hPSC-EMSCs elicit neuroprotective and neuroregenerative effects both *in vitro* and *in vivo* provide the possibility of utilising the stem cell secretome for HIE treatment in the future.

## Supplementary Material

Supplementary figures.

## Abbreviations

HIE: hypoxic-ischemic encephalopathy
MSCs: mesenchymal stromal cells
ESCs: embryonic stem cells
iPSCs: induced pluripotent stem cells
PSCs: pluripotent stem cells
hPSC-EMSCs: human pluripotent stem cell-derived ectomesenchymal stromal cells
hUC-MSCs: human umbilical cord-derived MSCs
NPCs: neural progenitor cells
PBS: phosphate-buffered saline
ELISA: enzyme-linked immunosorbent assay
ERK: extracellular signal-regulated kinase
CREB: cAMP response element-binding protein
CM: conditional medium
HI: hypoxia ischemic
NGF: nerve growth factor
PDGF: platelet-derived growth factor
TGF: transforming growth factor
CP: cerebral palsy
NC: neural crest
hNCPCs: human neural crest progenitor cells
cAMP: cyclic adenosine monophosphate
PRRH: peripherin
FGF: fibroblast growth factor
EGF: epidermal growth factor
BDNF: brain-derived neurotrophic factor
OGD/R: oxygen-glucose deprivation/reoxygenation
TUNEL: terminal transferase-mediated dUTP nick-end labeling
RPKM: reads per kilobase million
DEG: differentially expressed genes
FDR: false discovery rate
rBM-MSCs: rat bone marrow-derived mesenchymal stromal cells
IND: intranasal delivery
OCT: optimum cutting temperature compound
ROI: a region of interest
SVZ: subventricular zone
MWZ: Morris water maze
ANOVA: One-way analysis of variance
dpi: days post-injection
PKC: protein kinase C
PI3K: phosphoinositide 3-kinase
AD-MSCs: adipose tissue-derived MSCs
WJ-MSCs: Wharton's jelly-derived MSCs
ECM: extracellular matrix
BBB: blood-brain barrier
