# Supplementary material for: Human pluripotent stem cell-derived ectomesenchymal stromal cells promote more robust functional recovery than umbilical cord-derived mesenchymal stromal cells after hypoxic-ischaemic brain damage
Source: Theranostics. 2022 Jan 1;12(1):143–66. doi: 10.7150/thno.57234 (PMC8690936; doi:10.7150/thno.57234)

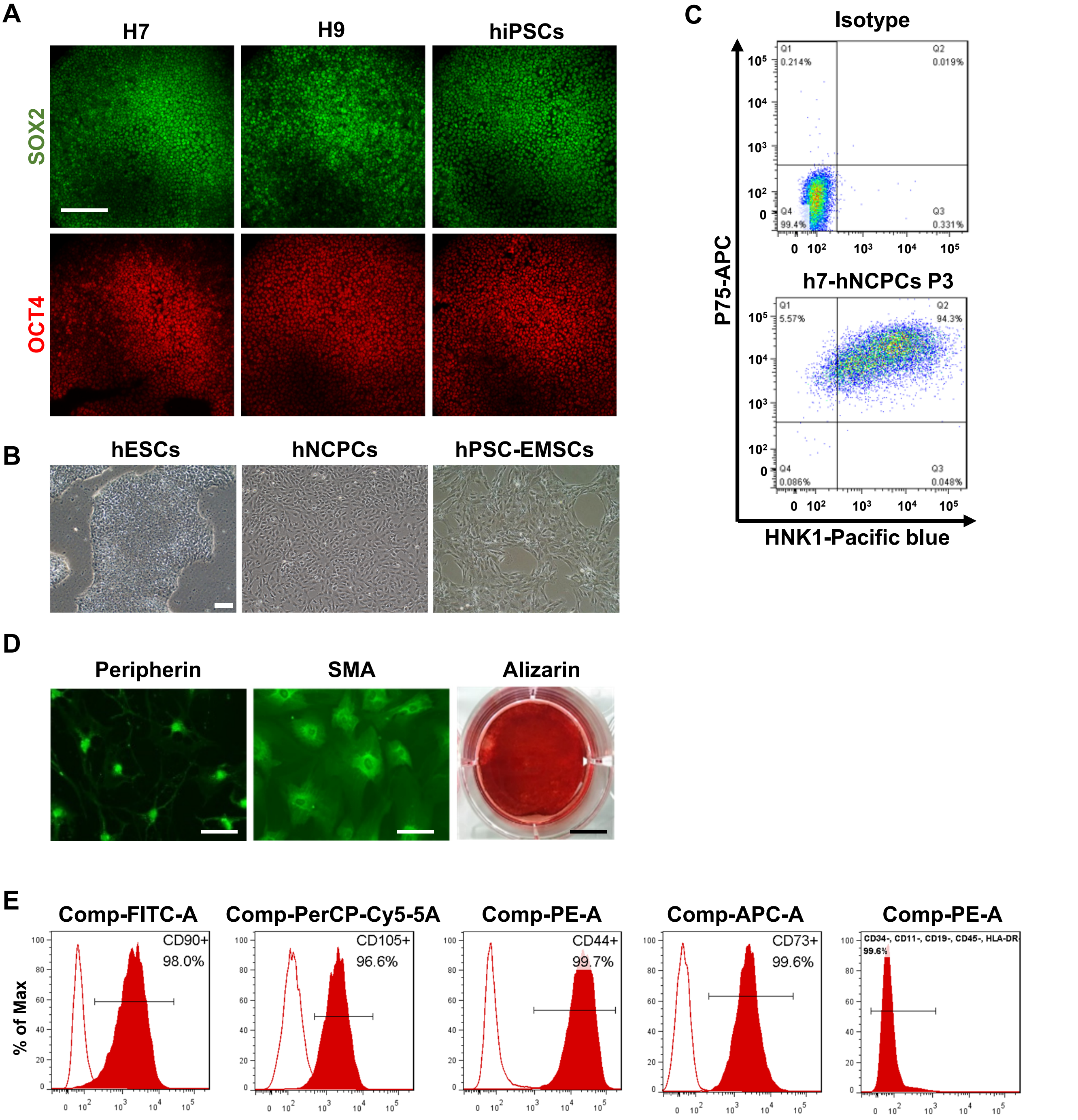
A
H7
H9
hiPSCs
SOX2
OCT4
B
hESCs
hNCPCs
hPSC-EMSCs
C
Isotype
P75-APC
h7-hNCPCs P3
HNK1-Pacific blue
Q1 0.214%
Q2 0.019%
Q4 99.4%
Q3 0.331%
Q1 5.57%
Q2 94.3%
Q4 0.086%
Q3 0.048%
D
Peripherin
SMA
Alizarin
E
Comp-FITC-A
Comp-PerCP-Cy5-5A
Comp-PE-A
Comp-APC-A
Comp-PE-A
% of Max
CD90+ 98.0%
CD105+ 96.6%
CD44+ 99.7%
CD73+ 99.6%
CD34-, CD11-, CD19-, CD45-, HLA-DR
99.6%

Figure S1: Characterization of hESC-derived EMSCs. A) Representative immunofluorescent staining of SOX2 and OCT4 in undifferentiated hESCs and hiPSCs, scale bar: 100 μm; **B)** Phase-contrast image of H7 hESCs, hNCPCs and hPSC-EMSCs, scale bar: 100 μm; **C)** Flow cytometry analysis of H7-derived hNCPCs using two neural crest markers, HNK1 and p75 after 16 days of differentiation from hESCs; **D)** hNCPCs were capable of differentiating into peripheral neurons, myofibroblasts and osteoblasts using the protocols described in the Methods section. Peripherin was used to stain the peripheral neurons (day 15) whereas SMA was used to stain myofibroblasts (day 30), scale bar: 100 μm; Alizarin Red was used to stain osteogenesis differentiation from hPSC-derived EMSCs (day 16), scale bar = 0.2 cm; **E)** Phenotypic characterization of H7-generated hPSC-EMSCs by MSC characterization kit using flow cytometry after 15 day-differentiation from hNCPCs. hPSC-EMSCs were positive for CD90, CD105, CD44 and CD73 and negative for hematopoietic markers, CD34, CD11, CD19, CD45 and HLA-DR. Experiments were repeated at least three times.

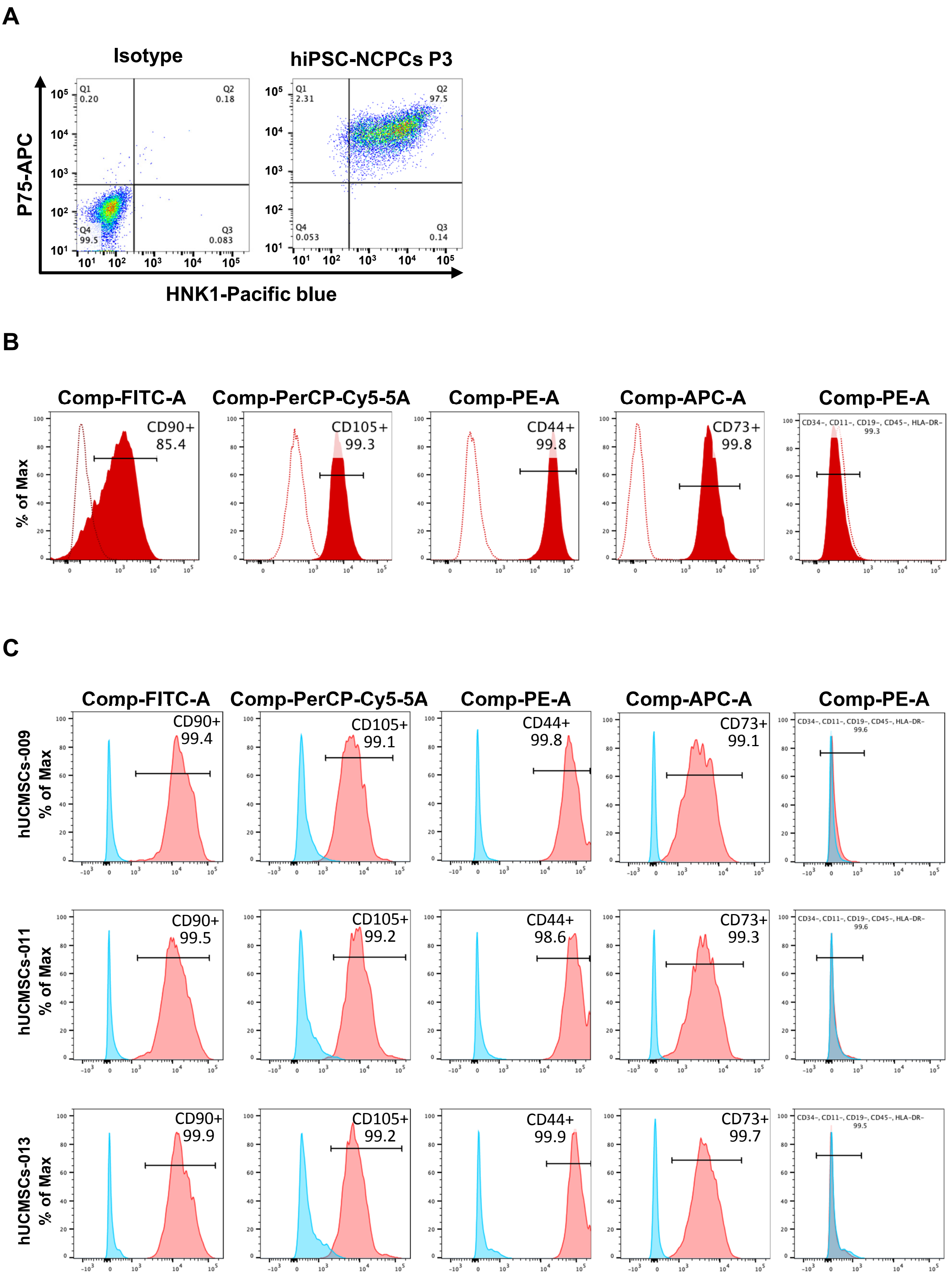
A
Isotype
hiPSC-NCPCs P3
P75-APC
HNK1-Pacific blue
Q1 0.20
Q2 0.18
Q4 99.5
Q3 0.083
Q1 2.31
Q2 97.5
Q4 0.053
Q3 0.14
B
Comp-FITC-A
Comp-PerCP-Cy5-5A
Comp-PE-A
Comp-APC-A
Comp-PE-A
% of Max
CD90+ 85.4
CD105+ 99.3
CD44+ 99.8
CD73+ 99.8
CD34−, CD11−, CD19−, CD45−, HLA-DR− 99.3
C
Comp-FITC-A
Comp-PerCP-Cy5-5A
Comp-PE-A
Comp-APC-A
Comp-PE-A
hUCMSCs-009
% of Max
CD90+ 99.4
CD105+ 99.1
CD44+ 99.8
CD73+ 99.1
CD34−, CD11−, CD19−, CD45−, HLA-DR− 99.6
hUCMSCs-011
% of Max
CD90+ 99.5
CD105+ 99.2
CD44+ 98.6
CD73+ 99.3
CD34−, CD11−, CD19−, CD45−, HLA-DR− 99.6
hUCMSCs-013
% of Max
CD90+ 99.9
CD105+ 99.2
CD44+ 99.9
CD73+ 99.7
CD34−, CD11−, CD19−, CD45−, HLA-DR− 99.5

Figure S2: Characterization of hiPSC-derived EMSCs. A) Flow cytometry analysis of hiPSC-derived hNCPCs using two neural crest markers, HNK1 and p75 after 16 days of differentiation from iPSCs. More than 95% of the hiPSC-derived hNCPCs expressed NCSC surface markers, p75 and HNK-1; **B)** FACS analysis of MSC surface marker expression in hiPSC-derived hPSC-EMSCs. The majority of the hiPSC-derived hPSC-EMSCs expressed MSC surface markers after 15 day-differentiation from hNCPCs, CD90 (85.4%), CD105 (99.3%), CD44 (99.8%) and CD73 (99.8%) and negative for hematopoietic markers, CD34, CD11, CD19, CD45 and HLA-DR; **C)** FACS analysis of MSC surface marker expression in three hUC-MSC lines. Experiments were repeated at least three times.

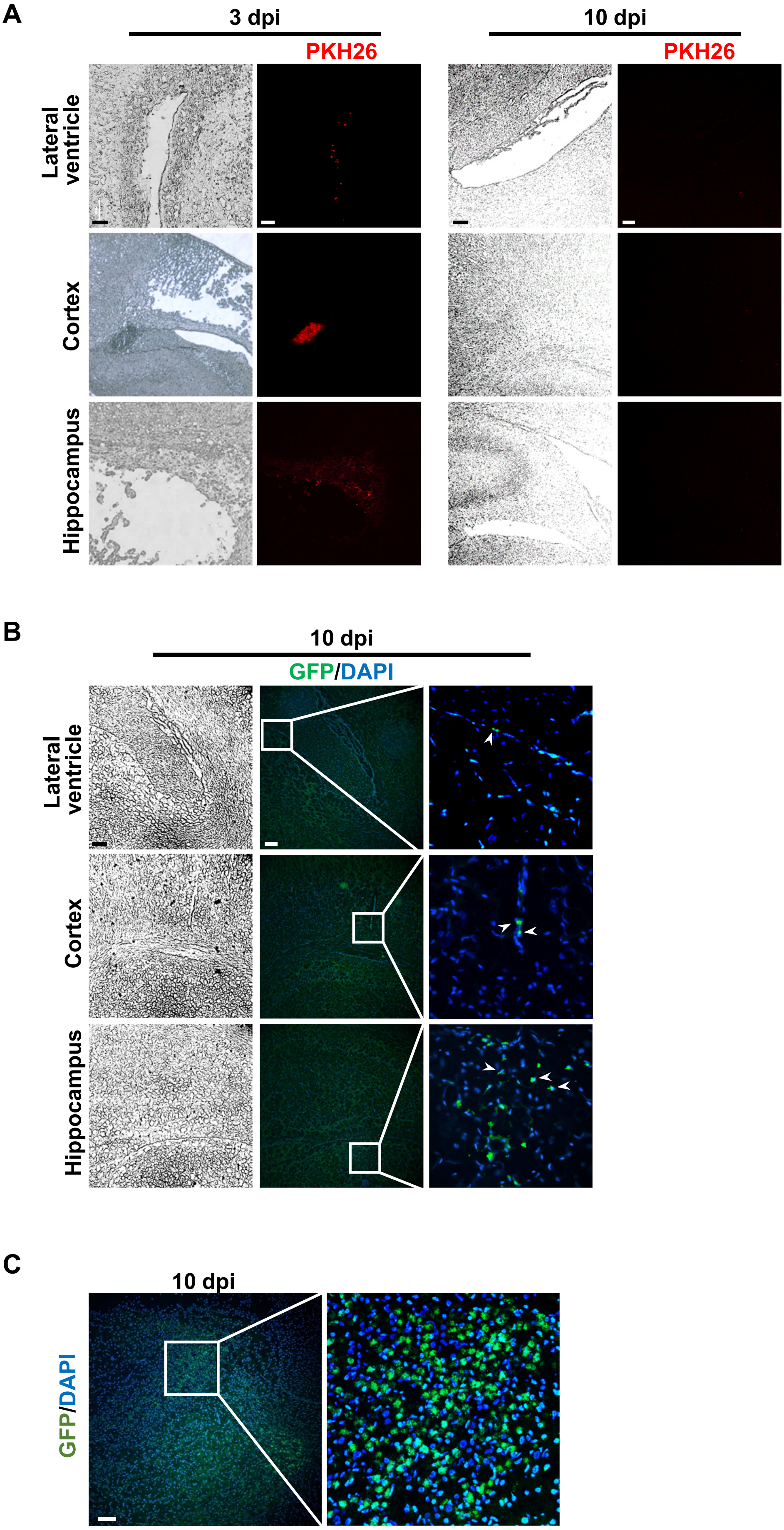
A
3 dpi
10 dpi
PKH26
PKH26
Lateral ventricle
Cortex
Hippocampus
B
10 dpi
GFP/DAPI
Lateral ventricle
Cortex
Hippocampus
C
10 dpi
GFP/DAPI

Figure S3: Detection of human and rat MSCs in the HIE rats. A) Visualization of transplanted PKH26 labelled hPSC-EMSCs in HIE rats, scale bar: 100 μm. Note that hPSC-EMSCs were detected in the lateral ventricle, cortex and hippocampus at 3 dpi, but not 10 dpi, n = 3; **B)** Visualization of transplanted GFP-labelled hPSC-EMSCs in HIE rats (10 dpi), scale bar: 100 μm. Note that only small number of hPSC-EMSCs were detected in the HIE brain (arrowhead), n = 3; **C)** $2x10^5$GFP-labelled rat BMSCs were transplanted 3 days after HI insult. Note that large amount of rat BMSCs was still detected in the striatum at 10 dpi, scale bar: 100 μm. n = 3.

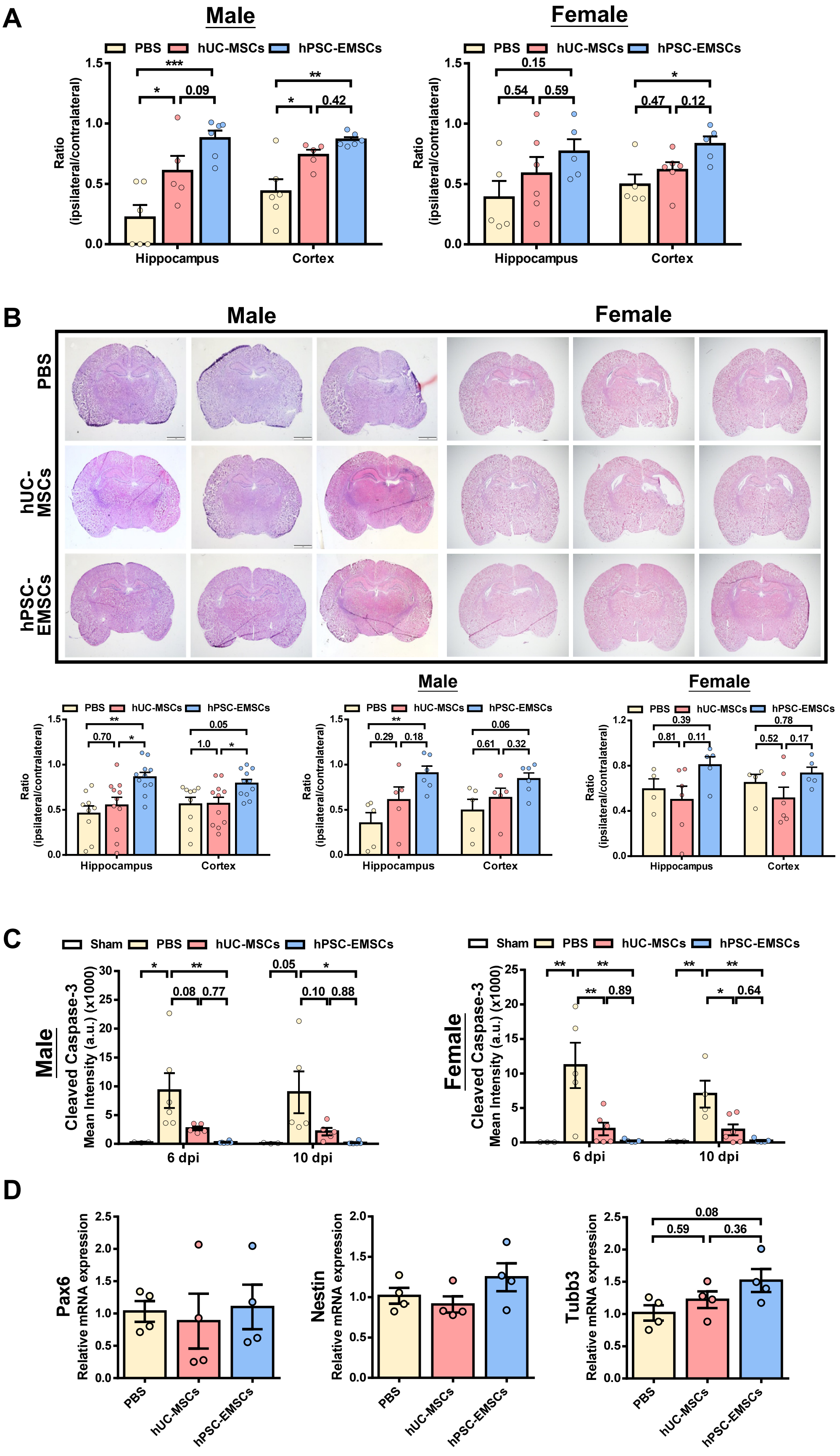
A
Male
Female
PBS
hUC-MSCs
hPSC-EMSCs
Ratio (ipsilateral/contralateral)
Hippocampus
Cortex
B
Male
Female
PBS
hUC-MSCs
hPSC-EMSCs
C
Sham
PBS
hUC-MSCs
hPSC-EMSCs
Male
Female
Cleaved Caspase-3 Mean Intensity (a.u.) (x1000)
6 dpi
10 dpi
D
Pax6
Nestin
Tubb3
Relative mRNA expression
PBS
hUC-MSCs
hPSC-EMSCs

Figure S4: hPSC-EMSCs promote brain injury repair in HIE rats. A) Images and quantification of H&E-stained ipsilateral/ contralateral brain cryosections collected from male and female rats at 6 dpi, scale bar: 2.5 mm. Quantification data represent mean ± SEM; **B)** Bright field images and quantification of H&E stained ipsilateral/ contralateral brain cryosections collected from male and female rats at 10 dpi, scale bar: 2.5 mm. Quantification data represented mean ± SEM; **C)** Quantification of cleaved caspase-3 expression in male and female rats at 6 and 10 dpi. The intensity was measured in three coronal sections (6 dpi, bregma -1.7, -1.8 and -1.9 mm; 10 dpi, bregma -2.0, -2.1 and -2.2 mm), scale bar: 100 µm. Quantification data represent mean ± SEM; **D)** Transplanted hPSC-EMSCs had no effects on mRNA expression levels of *nestin* and *Pax6* in the cortex of HIE rats, but showed a trend of upregulation in immature neuron marker, *Tubb3* (6 dpi, n = 4 rats per group). *, **, *** represent p < 0.05,0.01 and 0.001respectively by Tukey's *post-hoc* test when statistical significance by One-way ANOVA (p < 0.05) was obtained.

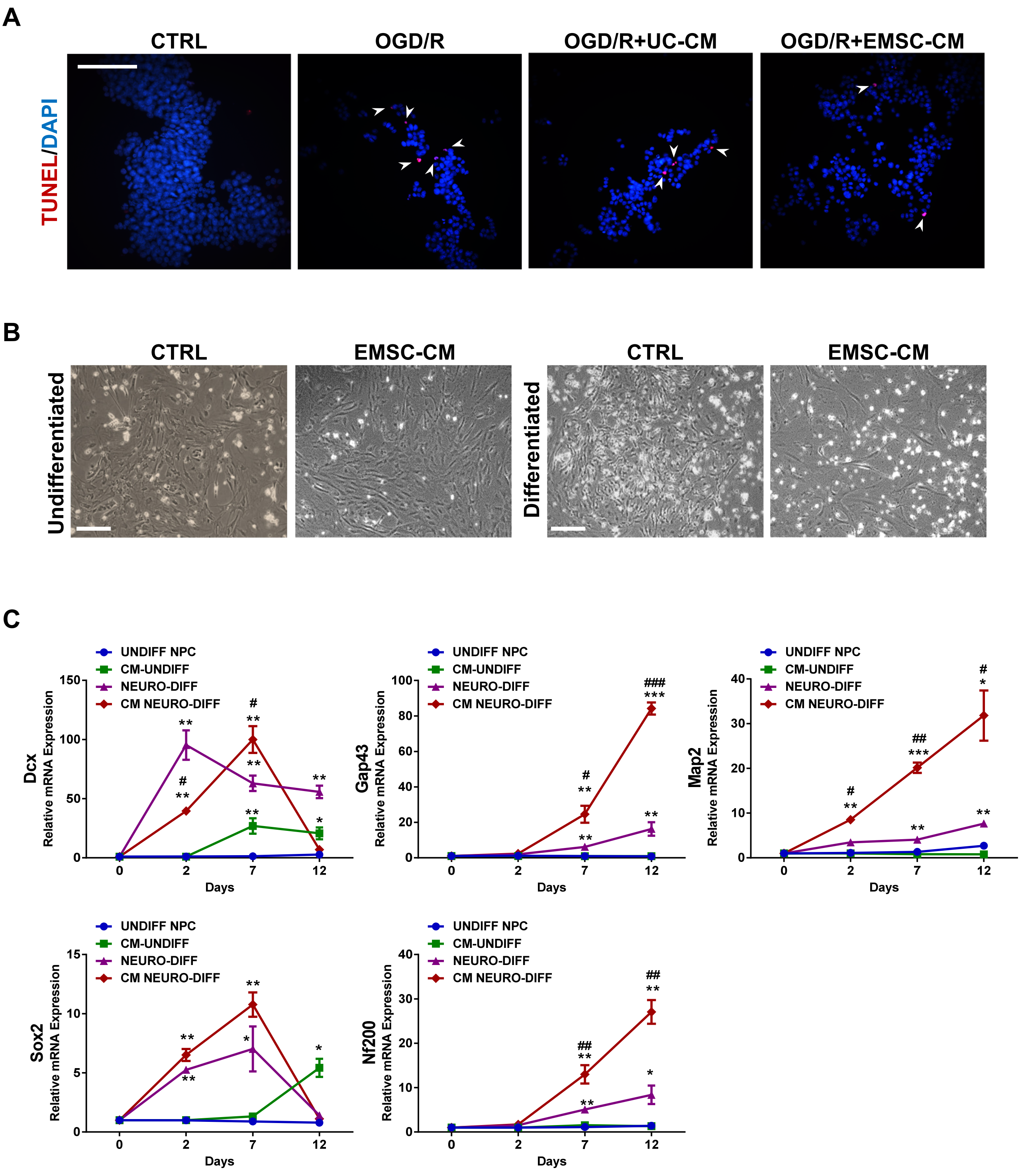
A
CTRL
OGD/R
OGD/R+UC-CM
OGD/R+EMSC-CM
TUNEL/DAPI
B
CTRL
EMSC-CM
CTRL
EMSC-CM
Undifferentiated
Differentiated
C
UNDIFF NPC
CM-UNDIFF
NEURO-DIFF
CM NEURO-DIFF
Dcx
Relative mRNA Expression
Days
Gap43
Map2
Sox2
Nf200

Figure S5: Effects of CM from hPSC-EMSCs on neurite outgrowth and neurogenesis. A) PC-12 cells were challenged with OGD/R (4 h OGD + 24 h re-oxygenation) with or without CM derived from either hPSC-EMSCs (hiPSCs) or hUC-MSCs. TUNEL assay was used to determine cellular apoptosis (white arrowhead), scale bar: 100 μm; **B)** Phase-contrast microscopy images of undifferentiated or differentiated primary rNPCs in the presence or absence of CM derived from hPSC-EMSCs, scale bar: 100 μm; **C)** rNPCs were induced to differentiate into neurons in the presence or absence of CM derived from hPSC-EMSCs for 12 days. Concurrent treatment of rNPCs with neuronal differentiation medium and EMSC- CM significantly elevated *Sox2*, *Dcx*, *Nf200, Map2* and *Gap43* gene expression in comparison to rNPCs treated with neuron differentiation medium only (NEURO-DIFF) or control (UNDIFF-NPC) alone. Quantification data represent mean ± SEM, *, **, *** represent $p < 0.05$, 0.01 and 0.001 respectively by Tukey's *post-hoc* test compared to UNDIFF-NPC when statistical significance by One-way ANOVA ($p < 0.05$) was obtained. $^{\#}$, $^{\#\#}$, $^{\#\#\#}$ represent $p < 0.05$, 0.01, 0.001 respectively by Tukey's *post-hoc* test compared to NEURO-DIFF group when statistical significance by One-way ANOVA ($p < 0.05$) was obtained.

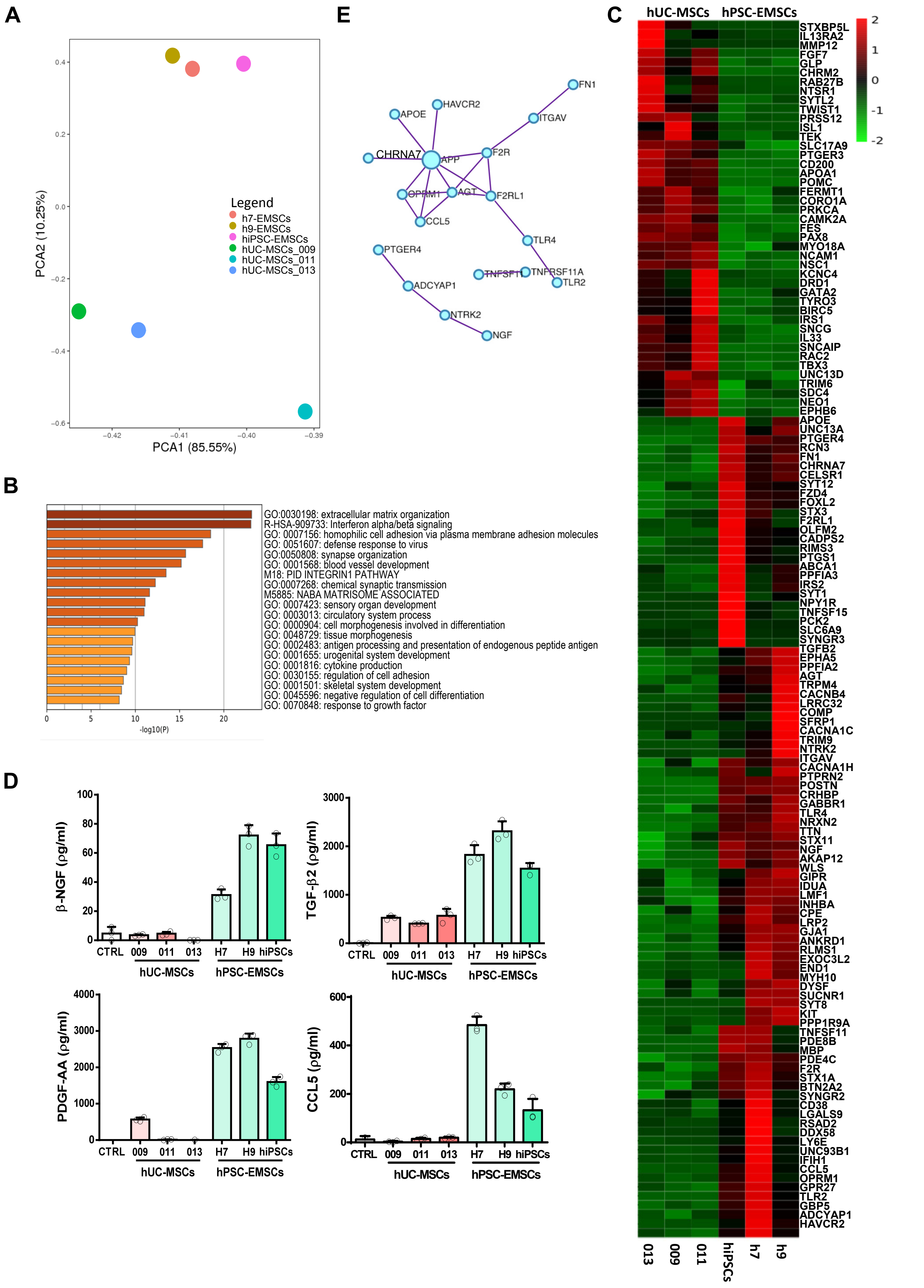

A
E
C
PCA2 (10.25%)
PCA1 (85.55%)
Legend
h7-EMSCs
h9-EMSCs
hiPSC-EMSCs
hUC-MSCs_009
hUC-MSCs_011
hUC-MSCs_013
FN1
HAVCR2
APOE
ITGAV
CHRNA7
APP
F2R
OPRM1
AGT
F2RL1
CCL5
TLR4
PTGER4
TNFSF11
TNFRSF11A
TLR2
ADCYAP1
NTRK2
NGF
hUC-MSCs
hPSC-EMSCs
STXBP5L
IL13RA2
MMP12
FGF7
GLP
CHRM2
RAB27B
NTSR1
SYTL2
TWIST1
PRSS12
ISL1
TEK
SLC17A9
PTGER3
CD200
APOA1
POMC
FERMT1
CORO1A
PRKCA
CAMK2A
FES
PAX8
MYO18A
NCAM1
NSC1
KCNC4
DRD1
GATA2
TYRO3
BIRC5
IRS1
SNCG
IL33
SNCAIP
RAC2
TBX3
UNC13D
TRIM6
SDC4
NEO1
EPHB6
APOE
UNC13A
PTGER4
RCN3
FN1
CHRNA7
CELSR1
SYT12
FZD4
FOXL2
STX3
F2RL1
OLFM2
CADPS2
RIMS3
PTGS1
ABCA1
PPFIA3
IRS2
SYT1
NPY1R
TNFSF15
PCK2
SLC6A9
SYNGR3
TGFB2
EPHA5
PPFIA2
AGT
TRPM4
CACNB4
LRRC32
COMP
SFRP1
CACNA1C
TRIM9
NTRK2
ITGAV
CACNA1H
PTPRN2
POSTN
CRHBP
GABBR1
TLR4
NRXN2
TTN
STX11
NGF
AKAP12
WLS
GIPR
IDUA
LMF1
INHBA
CPE
LRP2
GJA1
ANKRD1
RLMS1
EXOC3L2
END1
MYH10
DYSF
SUCNR1
SYT8
KIT
PPP1R9A
TNFSF11
PDE8B
MBP
PDE4C
F2R
STX1A
BTN2A2
SYNGR2
CD38
LGALS9
RSAD2
DDX58
LY6E
UNC93B1
IFIH1
CCL5
OPRM1
GPR27
TLR2
GBP5
ADCYAP1
HAVCR2
013
009
011
hiPSCs
h7
h9
B
GO:0030198: extracellular matrix organization
R-HSA-909733: Interferon alpha/beta signaling
GO: 0007156: homophilic cell adhesion via plasma membrane adhesion molecules
GO: 0051607: defense response to virus
GO:0050808: synapse organization
GO: 0001568: blood vessel development
M18: PID INTEGRIN1 PATHWAY
GO:0007268: chemical synaptic transmission
M5885: NABA MATRISOME ASSOCIATED
GO: 0007423: sensory organ development
GO: 0003013: circulatory system process
GO: 0000904: cell morphogenesis involved in differentiation
GO: 0048729: tissue morphogenesis
GO: 0002483: antigen processing and presentation of endogenous peptide antigen
GO: 0001655: urogenital system development
GO: 0001816: cytokine production
GO: 0030155: regulation of cell adhesion
GO: 0001501: skeletal system development
GO: 0045596: negative regulation of cell differentiation
GO: 0070848: response to growth factor
-log10(P)
D
β-NGF (ρg/ml)
TGF-β2 (ρg/ml)
PDGF-AA (ρg/ml)
CCL5 (ρg/ml)
CTRL
009
011
013
H7
H9
hiPSCs
hUC-MSCs
hPSC-EMSCs

Figure S6: Secretory factors are differentially expressed between hUC-MSCs and hPSC-EMSCs. A) hUC-MSCs and hPSC-EMSCs are two distinct cell populations by principal component Analysis (PCA); **B)** Gene ontology analysis of significant DEGs between hPSC-EMSCs and hUC-MSCs; **C**) Heatmap of 93 DEGs of cell secretion (GO: 0032940) that were differentially expressed between hPSC-EMSCs and hUC-MSCs; **D)** ELISA analysis of β-NGF, TGF-β_2, CCL5 and PDGF-AA in the CM derived from three hPSC-EMSCs lines and three hUC-MSCs lines. DMEM serves as control, experiments were repeated at least three times and quantification data represent mean ± SEM; **E)** The protein–protein interaction network for upregulated expression genes that were related to ERK1/2 cascade (GO: 0070371). The PPI network was produced using Metascape (http://metascape.org/).

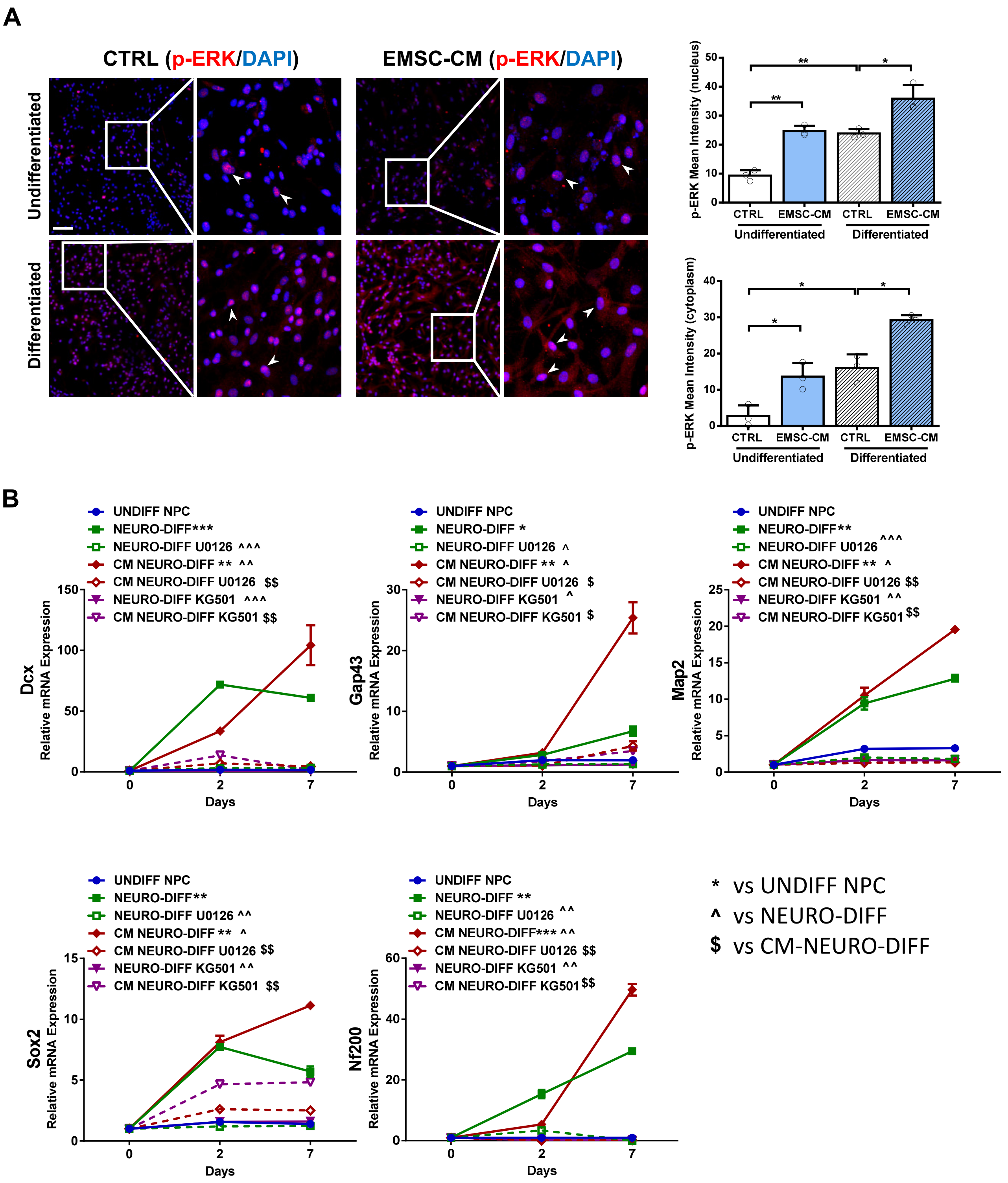
A
CTRL (p-ERK/DAPI)
EMSC-CM (p-ERK/DAPI)
Undifferentiated
Differentiated
p-ERK Mean Intensity (nucleus)
p-ERK Mean Intensity (cytoplasm)
CTRL
EMSC-CM
Undifferentiated
Differentiated
B
UNDIFF NPC
NEURO-DIFF***
NEURO-DIFF U0126 ^^^
CM NEURO-DIFF ** ^^
CM NEURO-DIFF U0126 $$
NEURO-DIFF KG501 ^^^
CM NEURO-DIFF KG501 $$
Dcx
Gap43
Map2
Sox2
Nf200
Relative mRNA Expression
Days
* vs UNDIFF NPC
^ vs NEURO-DIFF
$ vs CM-NEURO-DIFF

Figure S7: hPSC-EMSCs promotes neurogenesis via the ERK/CREB pathway. A) rNPCs were induced to differentiate into neurons with or without the CM derived from hPSC-EMSCs (hiPSCs) for 14 days. Focused area is shown with a magnified view(inset) illustrating p-ERK positive cells (red, arrowhead). Concurrent treatment of rNPCs with neuronal differentiation medium and EMSC-CM significantly increased the cytoplasmic and nuclear expression of p-ERK. Data shown is derived from three independent experiments. Five fields were analyzed per slide under the microscope and no less than 200 cells were analyzed for quantification data; Quantification data represent mean ± SEM, **represents $p < 0.01$ by Tukey's *post-hoc* test when statistical significance by One-way ANOVA ($p < 0.05$) is obtained; **B)** rNPCs were induced to differentiate into neurons in the presence or absence of CM derived from hPSC-EMSCs and either ERK or CREB inhibitor for 7 days. Concurrent treatment of rNPCs with neuronal differentiation medium and EMSC-CM significantly elevated *Sox2*, *Dcx*, *Nf200, Map2* and *Gap43* gene expression in comparison to rNPCs treated with neuron differentiation medium. Addition of either CREB or ERK inhibitor significantly alleviated the potentiation effects. Quantification data represent mean ± SEM, *, **, *** represent $p < 0.05$, 0.01 and 0.001 respectively by Tukey's *post-hoc* test compared to UNDIFF-NPC when statistical significance by One-way ANOVA ($p < 0.05$) was obtained. ^, ^^, ^^^ represent $p < 0.05$, 0.01, 0.001 respectively by Tukey's *post-hoc* test compared to NEURO-DIFF group when statistical significance by One-way ANOVA ($p < 0.05$) was obtained. $^{\$}$, $^{\$\$}$ represent $p < 0.05$, 0.01 respectively by Tukey's *post-hoc* test compared to CM-NEURO-DIFF when statistical significance by One-way ANOVA ($p < 0.05$) was obtained.

Full images of western blots for Figure 7A

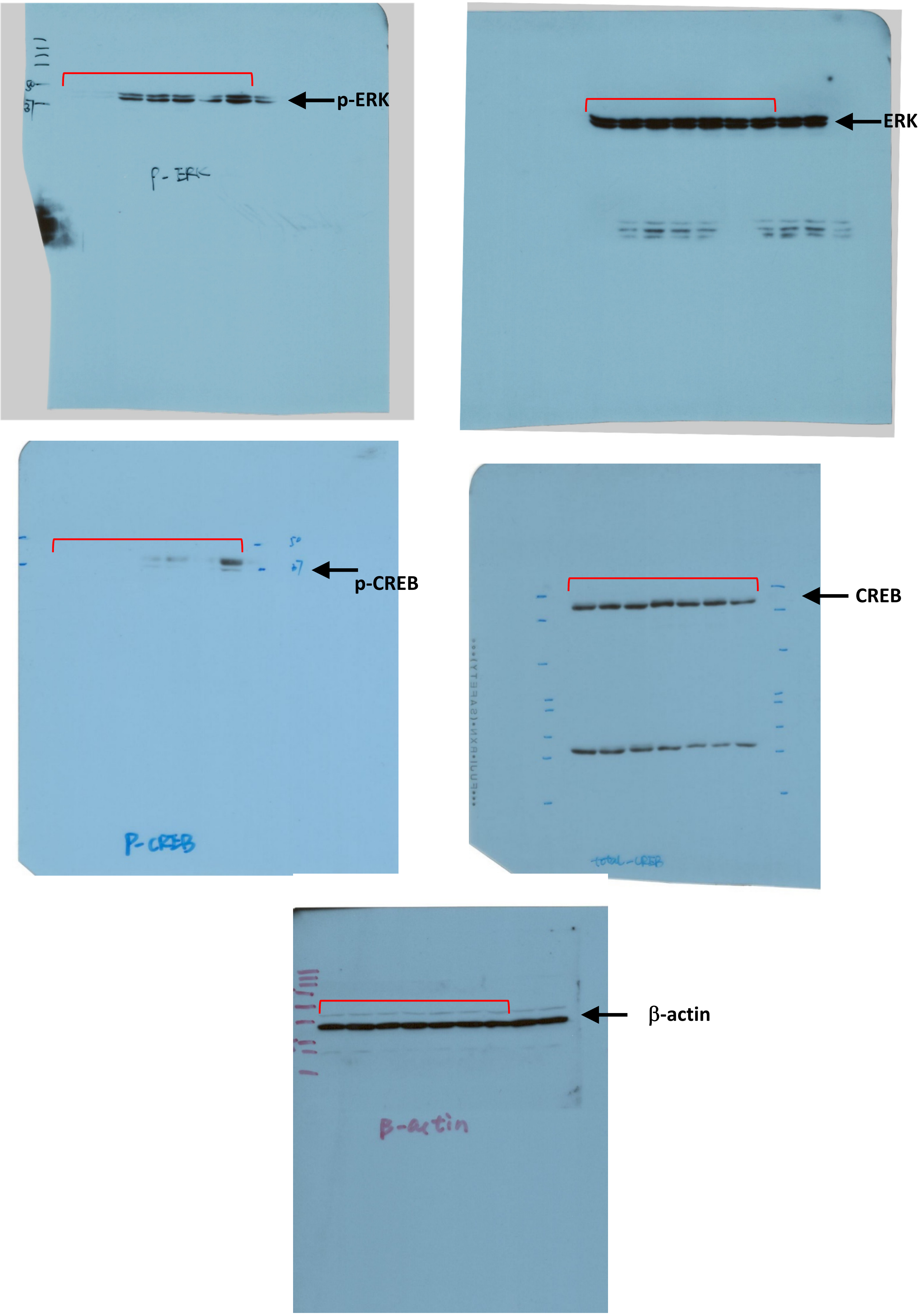

Full images of western blots for Figure 7B

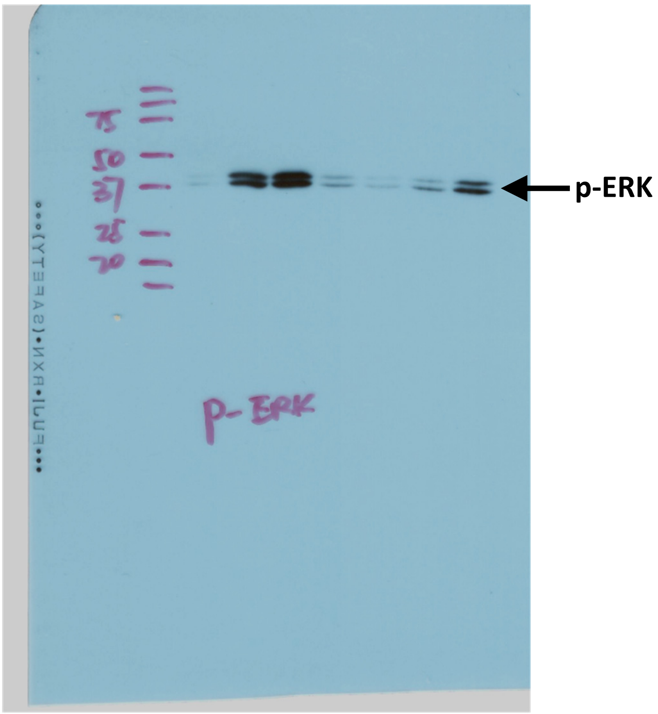

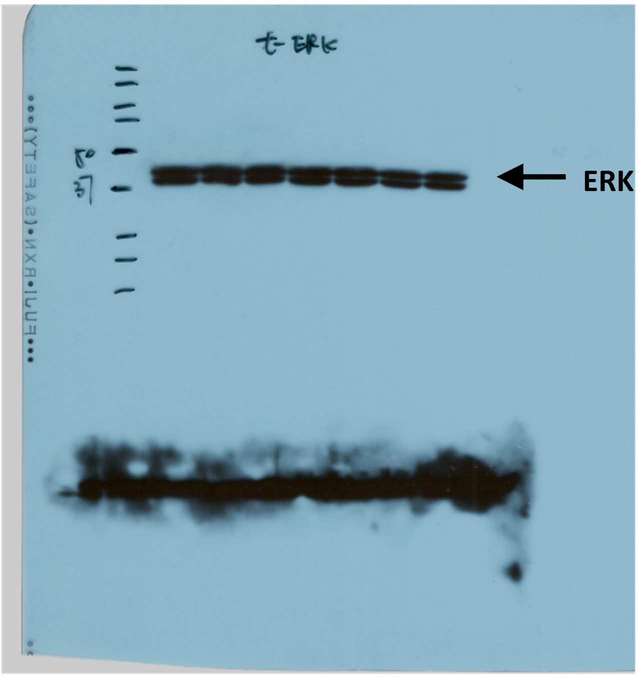

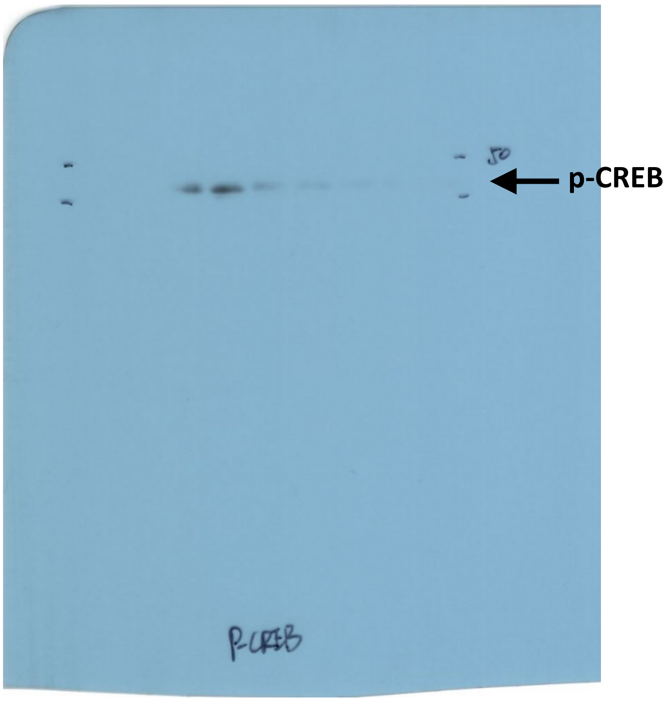

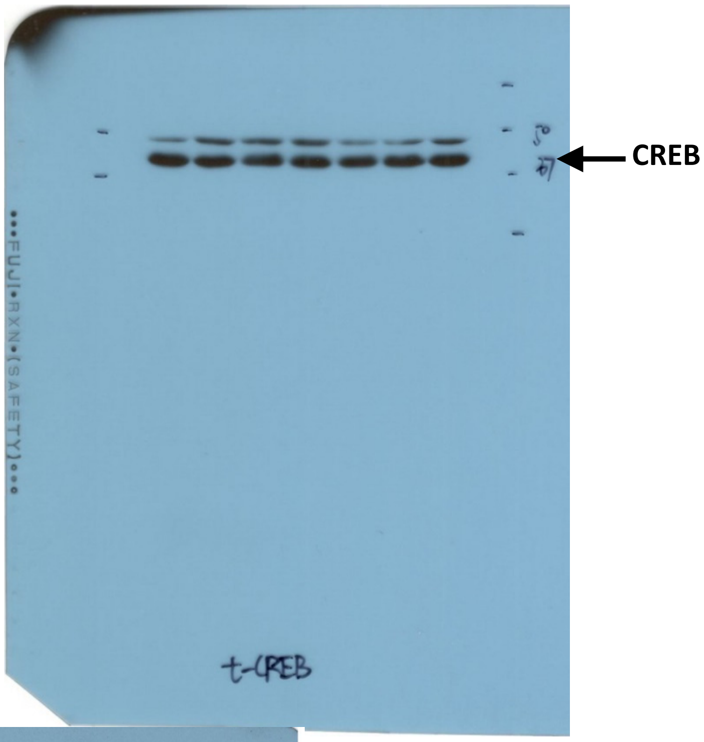

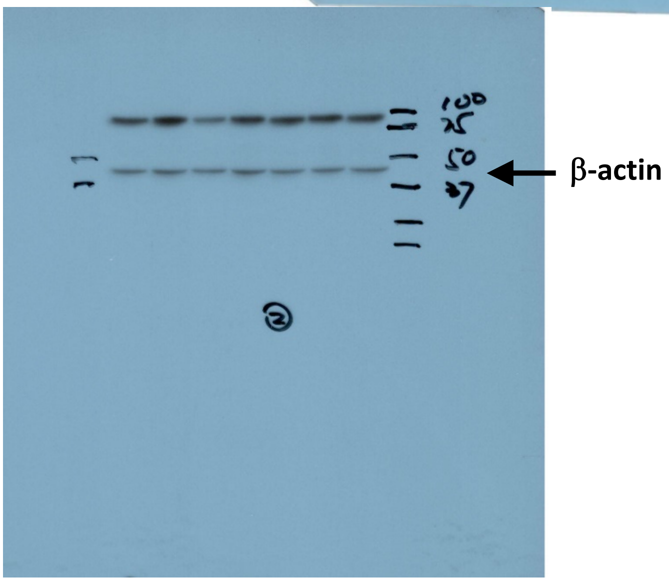

Supplement: Supplementary file 1 — Supplementary figures. [file thnov12p0143s1.pdf]
